# Endothelium and Subendothelial Matrix Mechanics Modulate Cancer Cell Transendothelial Migration

**DOI:** 10.1002/advs.202206554

**Published:** 2023-04-13

**Authors:** Yousef Javanmardi, Ayushi Agrawal, Andrea Malandrino, Soufian Lasli, Michelle Chen, Somayeh Shahreza, Bianca Serwinski, Leila Cammoun, Ran Li, Mehdi Jorfi, Boris Djordjevic, Nicolas Szita, Fabian Spill, Sergio Bertazzo, Graham K Sheridan, Vivek Shenoy, Fernando Calvo, Roger Kamm, Emad Moeendarbary

**Affiliations:** ^1^ Department of Mechanical Engineering University College London Torrington Place London WC1E 7JE UK; ^2^ Department of Biological Engineering Massachusetts Institute of Technology Cambridge MA 02139 USA; ^3^ 199 Biotechnologies Ltd Gloucester Road London W2 6LD UK; ^4^ Department of Biochemical Engineering University College London London WC1E 6BT UK; ^5^ School of Mathematics University of Birmingham Edgbaston Birmingham B152TS UK; ^6^ Department of Medical Physics and Biomedical Engineering University College London London WC1E 6BT UK; ^7^ School of Life Sciences Queen's Medical Centre University of Nottingham Nottingham NG7 2UH UK; ^8^ Department of Materials Science and Engineering University of Pennsylvania Philadelphia PA 19104 USA; ^9^ Instituto de Biomedicina y Biotecnología de Cantabria (Consejo Superior de Investigaciones Científicas, Universidad de Cantabria) Santander 39011 Spain; ^10^ Biomaterials, Biomechanics and Tissue Engineering Group Department of Materials Science and Engineering and Research Center for Biomedical Engineering Universitat Politécnica de Catalunya (UPC) 08019 Barcelona Spain

**Keywords:** biomaterial properties, cancer cell extravasation, computational modeling, metastasis, traction force microscopy

## Abstract

Cancer cell extravasation, a key step in the metastatic cascade, involves cancer cell arrest on the endothelium, transendothelial migration (TEM), followed by the invasion into the subendothelial extracellular matrix (ECM) of distant tissues. While cancer research has mostly focused on the biomechanical interactions between tumor cells (TCs) and ECM, particularly at the primary tumor site, very little is known about the mechanical properties of endothelial cells and the subendothelial ECM and how they contribute to the extravasation process. Here, an integrated experimental and theoretical framework is developed to investigate the mechanical crosstalk between TCs, endothelium and subendothelial ECM during in vitro cancer cell extravasation. It is found that cancer cell actin‐rich protrusions generate complex push–pull forces to initiate and drive TEM, while transmigration success also relies on the forces generated by the endothelium. Consequently, mechanical properties of the subendothelial ECM and endothelial actomyosin contractility that mediate the endothelial forces also impact the endothelium's resistance to cancer cell transmigration. These results indicate that mechanical features of distant tissues, including force interactions between the endothelium and the subendothelial ECM, are key determinants of metastatic organotropism.

## Graphical abstract

A combination of advanced engineering/material sciences, biological tools, and computational/ mathematical simulations are employed to study how the mechanical features of both endothelial cells and the subendothelial matrix at secondary organ sites may influence cancer metastasis and, in particular, the transendothelial migration of cancer cells, which is a major step during the extravasation process.

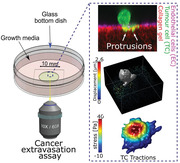

## 1 Introduction

A wide spectrum of genetic, biochemical, and mechanical factors drive the spread of tumor cells (TCs) to tissues and organs away from their primary locations, a process known as cancer metastasis.^[^ ^1^, ^2^, ^3^, ^4^ ^]^ The invasion of TCs into neighboring tissue, their intravasation into blood vessels^[^ ^5^ ^]^ and transport in the vascular system, followed by their extravasation and proliferation at distant sites are the major steps of metastatic dissemination.^[^ ^6^, ^7^ ^]^ The latter steps of metastasis involving survival in circulation, intravascular interactions, and transendothelial migration (TEM) are influenced by organ‐specific cues and, therefore, TCs from a certain primary tumor tend to metastasize to specific distant organs.^[^ ^8^, ^9^ ^]^ Such organ‐specific patterns of TC dissemination (i.e., organotropism) were conceptualized in the “seed and soil” hypothesis which describes the metastatic sites as favorable hosting environments^[^ ^10^ ^]^ that must provide appropriate biochemical and mechanical features for successful extravasation and colonization.

The role of different molecular players and cell types on organotropism has been extensively investigated,^[^ ^11^, ^12^, ^13^, ^14^ ^]^ but the impact of physical forces and mechanical features on extravasation has been overlooked mainly due to the relative scarcity of suitable methods to probe extravasation mechanics. For example, in transwell assays, the most widely used in vitro model for investigating TC extravasation, the effects of the subendothelial extracellular matrix (ECM) are typically ignored and it is not possible to study the mechanical functions of the endothelium and subendothelial ECM on TEM.^[^ ^15^ ^]^ On the other hand, in vivo studies in mouse,^[^ ^16^ ^]^ zebrafish,^[^ ^17^ ^]^ and chick embryo^[^ ^18^ ^]^ recapitulate more realistic environments at the extravasation sites,^[^ ^19^ ^]^ but it is extremely difficult to probe mechanical features, such as force interactions, with these approaches.^[^ ^20^ ^]^

The process of extravasation is initiated by physical trapping and/or adhesion of circulating TCs in the small vessels of distant organs and development of invasive protrusions^[^ ^21^ ^]^ that interact with endothelial cells (ECs) at their junctions, leading to the opening of endothelial gaps.^[^ ^22^ ^]^ Following initial gap opening, TCs must maintain the opening and expand the gap size to transmigrate through it. Therefore, successful TEM depends on both the ability of circulating TCs to generate forces and withstand large deformations, and the mechanical integrity of the endothelium of the remote tissues which, in turn, relies on actin polymerization and actomyosin contractility in the ECs.^[^ ^23^, ^24^ ^]^ Actin dynamics are mediated by intracellular signals,^[^ ^25^ ^]^ including RhoA‐ROCK pathways,^[^ ^26^, ^27^, ^28^ ^]^ and also extracellular mechanical cues, such as ECM stiffness.^[^ ^24^ ^]^ Indeed, the ECs forming the microvasculature at distant tissues sense and respond to tissue mechanical properties through modulation of their Rho‐GTPase signaling balance and actomyosin contractility.^[^ ^29^, ^30^ ^]^ Consequently, it is highly plausible that the mechanical characteristics of distant organs can impact TC extravasation partly through the biomechanical features of organ‐specific endothelium. For example liver, which is extremely susceptible to metastasis,^[^ ^31^, ^32^ ^]^ has a highly fenestrated microvasculature^[^ ^33^, ^34^ ^]^ with a weak basement membrane and a soft ECM.^[^ ^35^ ^]^ On the other hand, skin is one of the least preferable metastatic sites^[^ ^32^ ^]^ and has one of the most robust microvasculature^[^ ^36^ ^]^ and ECM.^[^ ^37^ ^]^

Motivated by the significance of biomechanical mechanisms involved during organotropism and particularly extravasation, we developed a 3D functional assay that offers several advantages including high resolution live‐imaging of extravasation events and the measurement of force interactions between cells and subendothelial ECM. Furthermore, through integration of experimental data with computational modeling, we unraveled the role of mechanical crosstalk between ECs, TCs, and ECM as well as the impact of the mechanical properties of subendothelial ECM on TEM mechanics. We propose that mechanical features of the endothelium and subendothelial ECM may represent a novel predictor of the degree of TC extravasation and metastatic potential, with relevant implications in understanding organotropic dissemination.

## 2 3D Assay for Probing Extravasation Mechanics

**Figure 1:**
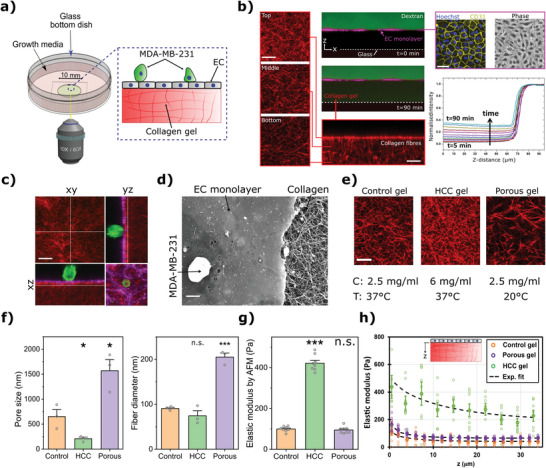
3D Extravasation assay for probing mechanics of cancer cells, endothelium, and the underlying ECM. a) Schematic representation of the extravasation assay. b) Quantification of the permeability of the HUVECs monolayer (magenta) formed on top of a thin collagen substrate. The permeability was calculated by measuring the changes of 70 kDa dextran (green) intensity in the collagen region using time‐lapse confocal imaging. Right images show the ECs intercellular junctions (CD31‐Yellow) and nuclei (Hoechst‐blue). Left and bottom images show the compaction of collagen fibers (red) due to the formation of endothelial monolayer. Scale = 20 µm. c) Confocal image of an MDA‐MB‐231 (cytoplasmic green), HUVECs (F‐actin magenta), and collagen fibers (red) taken by a 60× objective. Scale = 20 µm. d) Scanning electron microscopy image of the MDA‐MB‐231, HUVECs monolayer, and collagen fibers. Scale = 10 µm. e) Confocal images of the top layer of collagen (located underneath the washed off EC monolayer) for control, HCC, and porous collagen conditions. Scale = 20 µm. f) Quantification of the pore size and thickness of collagen fibers in different collagen gel conditions (mean ± S.E.M., *n* = 3 samples, in each sample (dish) 3 randomly selected regions were imaged using a scanning electron microscope; data points represent the average pore size/fiber thickness calculated for each dish). g) Elastic modulus of the collagen gels measured by atomic force microscopy indentation tests on the top surface of the gels after washing the EC monolayer (mean ± S.E.M., *n* = 8 dishes. In each dish at least 20 measurements were carried out. Data points represent average elastic modulus in each dish). h) Elastic modulus as a function of distance from the top surface for the three collagen gel conditions (mean ± S.E.M., *n* = 8 dishes. Each data point represents the average elastic modulus of the gel (dish) at a specific depth, calculated via the procedure explained in Note S2, Supporting Information). (For panels (f) and (g): *p < 0.05 and **p < 0.01, Student's *t*‐test).

To study the mechanics of TC extravasation and unravel the role of endothelium mechanics, we developed an assay that consists of human umbilical vein endothelial cells (HUVECs) cultured on a thin (≈100 µm) layer of collagen gel, which over the course of 48 h forms a tight endothelial monolayer (**Figure** **1**a,b). Staining of intercellular junctions with CD31 indicated the integrity and tightness of the EC monolayer formed in our assay (Figure 1b). Collagen gelmimics physiologically relevant ECM and, contrary to basement membrane‐based matrices, they form a tight network of fibers amenable to microscope‐based characterization. In addition, they allow for experimental tuning of physical and chemical characteristics by employing different crosslinking conditions or collagen concentrations. Finally, the porous nature of collagen gel enabled us to measure the permeability of the endothelium by introducing fluorescent dextran on top of the EC monolayer and monitoring the rate of change in fluorescence intensity inside the gel, using time‐lapse confocal microscopy (Figure 1b). 70 kDa dextran slowly passed through the tight EC monolayer and infiltrated into the void spaces between collagen fibers over time, leading to a gradual increase in dextran intensity within the gel. Measurements of dextran intensity variations over 90 min indicated the permeability of EC monolayer (0.8 ± 0.25 x10^−7^ cm s^−1^) to be similar to in vitro and in vivo values reported previously.^[^ ^38^ ^]^

Following formation of the tight physiological endothelial monolayer, TCs were introduced on top on the endothelium to investigate their interactions with the ECs and the compliant subendothelial collagen substrate, using live confocal microscopy (Figure 1c). Low magnification objectives (10× and 20×) were used to study the macroscopic events associated with cancer cell extravasation (i.e., TEM rate, duration, and efficiency) while images acquired with high magnification objectives (40× and 60×) were employed to probe dynamics of TC protrusion and collagen deformations. Scanning electron microscope images of the assay further indicated the integrity of the endothelial monolayer and the structure of subendothelial ECM (Figure 1d).

### 2.1 Mechanical Characterization of the Subendothelial Collagen Matrices

To perturb the mechanical and structural properties of the subendothelial ECM (mimicking different mechanical properties of the secondary tissues) and investigate their impact on TC extravasation, we used two collagen concentrations and two curing temperatures to make three different collagen gels: control (2.5 mg mL^−1^, 37 °C), highly porous (2.5 mg mL^−1^, 20 °C), and high collagen content (HCC, 6 mg mL^−1^, 37 °C). After collagen polymerization, ECs were added on top and allowed to form a tight connected monolayer over two days. During formation of the endothelial monolayer, ECs generated a basement membrane and significantly remodeled the collagen fibers^[^ ^39^ ^]^ causing an increase in density of the fibers near the top surface of the matrix (Figure 1b).Therefore, we quantified the physical properties of the remodeled collagen gel (Figure 1e) after washing off the EC monolayer (by Triton X treatment).

Scanning electron microscopy images of the collagen (Supplementary Note 1, Figures S1 and S2, Supporting Information) showed that while pore size of the porous condition (1.57 ± 0.22 µm) was significantly larger than that of the control gel (0.65 ± 0.14 µm, *p*‐value = 0.024 compared to porous gel), the pore size of the HCC was significantly less than that for control gels (0.21 ± 0.03 µm, *p*‐value = 0.035 compared to control, Figure 1f). Additionally, the fiber thickness of the HCC and control gels appeared to be in the same range (74 ± 11 and 91 ± 3 nm, respectively, *p*‐value = 0.24) while it was significantly larger for the porous gel (205 ± 9 µm, *p*‐value = 2.5 × 10^‐4^ compared to control, Figure 1f).

To better characterize the physical properties of the collagen hydrogels, we designed a new experiment to measure the permeability of control and porous gels (Note S1, Figure S3a, Supporting Information). Following the gelation of collagen inside a small channel, infiltration of a dye along the channel was measured over time. These measurements revealed permeability of the porous gel (0.015 cm min^−1^) to be almost twice as large as the control gel (0.008 cm min^−1^; *p*‐value = 1.3 × 10^−4^, Figure S3b,c, Supporting Information).

Next, atomic force microscopy (AFM) indentation tests on the top of the decellularized collagen gels (Figure 1g, Figure S4, Supporting Information) revealed over three‐fold higher elastic modulus for the HCC condition compared to the control (422 ± 14 Pa vs 100 ± 4 Pa, *p*‐value = 2 × 10^−12^) and no significant changes in the elastic modulus for the porous gel (95 ± 6 Pa, *p*‐value = 0.502, compared to control, Figure 1g). These measurements are consistent with our characterization of collagen structural properties (Figure 1f) showing the effects of fiber thickness and void size: thicker fibers tend to increase the stiffness while larger pore size leads to reduced stiffness.^[^ ^40^ ^]^ Since the porous condition exhibit both larger pore size and fiber thickness compared to the control condition, the elastic moduli of the porous and control gels are in the same range. On the other hand, the HCC condition has smaller pore size, but similar fiber thickness compared to the control leading to a significantly larger stiffness.

Finally, to account for the inhomogeneous mechanical properties as the result of EC‐induced collagen remodeling, we estimated elastic modulus as a function of depth (Figure 1h). This was achieved by knowing the elastic modulus measured by AFM (Figure 1g) and correlating the depth‐dependent elastic modulus with the pore size of the collagen at each depth as measured from confocal z‐stack images (see Note S2 and Figures S5–S9, Supporting Information).

Taken together, we developed a functional assay that incorporates an endothelium with physiologically relevant barrier function. This approach has the major advantage of conducting 3D high‐resolution spatiotemporal imaging of EC and TC behavior during extravasation. It also enables the quantification of EC or TC generated forces by live imaging of the collagen dynamics within gels. Furthermore, ECM properties can be tuned to have stiffnesses similar to human tissues, such as breast, bone marrow, and brain, which are all common sites of breast cancer extravasation.^[^ ^41^, ^42^, ^43^, ^44^ ^]^

### 2.2 The Role of Mechanical Signals on EC Generated Forces

**Figure 2:**
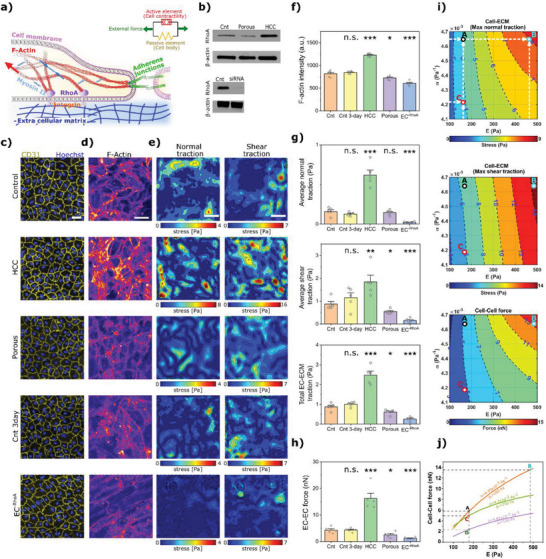
Collagen mechanical properties modulate mechanics of endothelial monolayer. a) Schematic of pathways involved in modulation of endothelium forces. The cytoskeleton of ECs is simulated using a chemo‐mechanical model that has an active element acting in parallel with a passive element representing cell stiffness. b) Levels of RhoA expression in ECs, for monolayers formed on control, high collagen content (HCC), and porous substrate (top), as well as siRNA RhoA knockdown in ECs (bottom). c) Examination of the integrity of the EC monolayer by intercellular junctional staining (CD31 in yellow and nuclei by Hoechst in blue) for different collagen conditions, culture‐time post seeding on collagen (Cnt 3‐day) and siRNA RhoA knockdown in ECs. Scale = 30 µm. d) Effects of different conditions on ECs F‐ actin organization and expression. Scale = 50 µm. e) Incremental normal and shear traction forces generated by the endothelial monolayer in different cases. Scale = 50 µm. f) Quantification of intensity of F‐actin for different conditions (mean ± S.E.M., *n* = 5 dishes. In each dish seven randomly chosen ROIs were imaged using confocal microscopy and each data point represents the average intensity value for each dish). g) Average cumulative total, normal, and shear tractions applied by the EC monolayer to ECM in different conditions (mean ± S.E.M., *n* = 5 dishes. In each sample (dish) three randomly chosen ROIs were imaged using a confocal microscope. Data points represent the average of traction forces for each dish). h) Comparison of magnitude of net tugging forces applied to an EC by its adjacent ECs (mean ± S.E.M., *n* = 5 dishes. In each sample (dish) three randomly chosen ROIs were imaged using a confocal microscope. Data points represent the average of tugging forces for each dish). For panels (f)–(h), *p < 0.05, **p < 0.01, and ***p < 0.001, Student's *t*‐test. i) Simulations show the impact of stiffness of the substrate (*E*) and its porosity (feedback parameter *α*) on the normal and shear traction forces applied by EC monolayers to the substrate, and on tugging forces. j) Effects of *E*, *α*, and levels of RhoA dependent contractility (*ρ*_0_) on the tugging forces of ECs. The points A, B, C, and D correspond to experimental data for control, HCC, porous, and EC^−RhoA^ cases, respectively.

Having quantified the mechanical properties of different subendothelial matrices, we investigated how they impacted the ability of ECs, integrated in a monolayer, to generate forces. Furthermore, we examined the role of RhoA as a key mechanobiological signaling molecule with critical roles in the regulation of cell–ECM and cell–cell mediated forces via actomyosin contractility^[^ ^45^ ^]^ (**Figure** **2**a). Notably, the expression of RhoA in ECs was upregulated when the monolayer was formed on the stiff (HCC) gel (Figure 2b), which is consistent with previous studies.^[^ ^46^, ^47^ ^]^ Moreover, RhoA expression in ECs on the porous gel was significantly less than on the control substrate (*p*‐value = 0.0015, Figure 2b), implying that matrix porosity also affects EC‐ECM interactions and downstream mechanotransduction. Considering that the mechanical changes in substrate influence RhoA signaling in ECs, we also directly downregulated (using siRNA knockdown) RhoA in ECs (indicated by EC^−RhoA^) after the formation of the monolayer on the control gel (Figure 2b and Figure S10, Supporting Information, see Section 4).

Next we assessed the impact of mechanical cues and RhoA signaling perturbations on EC junctional integrity and, in particular, on actin structures that are the key generators of cell–cell and cell–ECM contractile forces.^[^ ^48^ ^]^ Staining EC junctions using CD31 revealed the full integrity of the endothelium in all cases (Figure 2c, Video S1, Supporting Information). Notably, F‐actin intensity increased significantly in the HCC gel scenario compared to control (*p*‐value = 1.1 × 10^−45^), whereas it decreased significantly for porous and EC^−RhoA^ cases (*p*‐value = 0.015 and 1.7 × 10^−16^, respectively) (Figure 2d,f). Similar findings were observed when we stained for myosin II activity, MLC2, and pMLC2 (Note S3, Figure S11, Supporting Information): endothelial monolayers formed on HCC gel had significantly higher ratio of pMLC2/MLC2 compared to control (0.81 and 0.55, respectively, *p*‐value = 5.4 × 10^−4^) while this ratio was significantly less for the porous case (*p*‐value = 0.025 compared to control). We also extended the EC culture on the control collagen gel by one day (called Cnt 3‐day with 72 h culture time instead of 48 h) to investigate the influence of culture duration on endothelium and its interactions with ECM. EC monolayers in Cnt 3‐day condition did not show any significant changes in F‐actin expression (*p*‐value = 0.715, Figure 2d,f) compared to control. Interestingly, confocal z‐stack images of collagen fibers revealed similar structures for the control, Cnt 3‐day, and EC^−RhoA^ cases and therefore we considered same mechanical properties for these conditions (Figure S9, Supporting Information).

We assessed the influence of the above perturbations on EC‐ECM forces by employing traction force microscopy. Time‐dependent 3D deformation fields were measured by running a fast‐iterative digital volume correlation (FIDVC) algorithm^[^ ^49^ ^]^ on the xyz‐t stacks (taken every 15 min) of collagen gels deformed by ECs. The quantified strain fields were translated into stress fields while considering the depth‐dependent mechanical behavior of the subendothelial matrices (Figure 1e). Interestingly, perturbations in collagen matrix properties or in the activity of RhoA in ECs changed normal, shear, and total traction fields (Figure 2e). Particularly, the endothelial monolayers cultured on HCC gels generated the highest level of stresses (2.8‐fold increase in total force compared to control, *p*‐value = 7.1 × 10^−5^, Figure 2g), while downregulating RhoA in ECs led to the lowest level of traction forces (71% decrease in total force compared to control, *p*‐value = 1.76 × 10^−4^, Figure 2g). Culturing the ECs on porous matrix also decreased the tractions by 29% compared to control (*p*‐value = 0.038, Figure 2g). Increasing the culture time of ECs from 48 to 72 h did not change the total stress (*p*‐value = 0.22).

Finally we quantified intercellular forces that are applied to an EC by its adjacent ECs, that is, mechanical tugging force,^[^ ^50^ ^]^ by assuming that the monolayer is in mechanical equilibrium at each time step (quasi‐static condition, see Figure S12, Supporting Information). The effects of different perturbations on EC–EC forces showed similar trend as EC‐ECM traction forces (Figure 2h). The stiff matrix (HCC case) led to a 3.78‐fold increase in EC‐EC intercellular forces compared to control (*p*‐value = 3.8 × 10^−4^, Figure 2h). Downregulation of RhoA or using a porous matrix led to a 73% decrease (*p*‐value = 5.0 × 10^−4^) and 41% decrease (*p*‐value = 0.036) in EC–EC forces, respectively. Finally, for the Cnt 3‐day case, no significant change in EC‐EC forces was observed (*p*‐value = 0.98). Taken together, our data showed a direct correlation between the stiffness of subendothelial ECM, level of RhoA expression and abundance of F‐actin stress fibers in ECs, and the ability of ECs to generate traction or tugging forces.

### 2.3 Predictive Model of ECs Mechanics

To better understand the mechanisms regulating EC–EC and EC–ECM mechanical interactions, we developed a computational finite element model in which a linear elastic model was used to describe the subendothelial ECM and the EC nuclei. For EC cytoskeletons, we employed a model developed by Shenoy et al.^[^ ^51^ ^]^ which was capable of simulating the EC–ECM interaction (Figure 2a). Thus, the effects of changes in matrix stiffness on EC contractility and force generation could be readily evaluated. The effects of matrix porosity can also be captured by changing the feedback parameter *α*, so that less porous matrices were assigned higher *α* values, while motor density in quiescent state *ρ*_0_ was used to accommodate the effect of cells contractility potential (see Note S4 and Figure S13, Supporting Information for details on the model). Consistent with our experimental data (Figure 2g), the model predicted higher levels of normal and shear tractions and tugging forces when either the stiffness of ECM or the feedback parameter increased (Figure 2i, with points A, B, and C corresponding to the control, HCC, and porous cases, respectively). Furthermore, our simulation indicated that suppressing cell contractility by reducing *ρ*_0_ leads to a decrease in EC–EC forces (Figure 2j), with point D corresponding to EC^−RhoA^ case.

### 2.4 Extravasation Dynamics and Forces During Tumor Cell TEM

**Figure 3:**
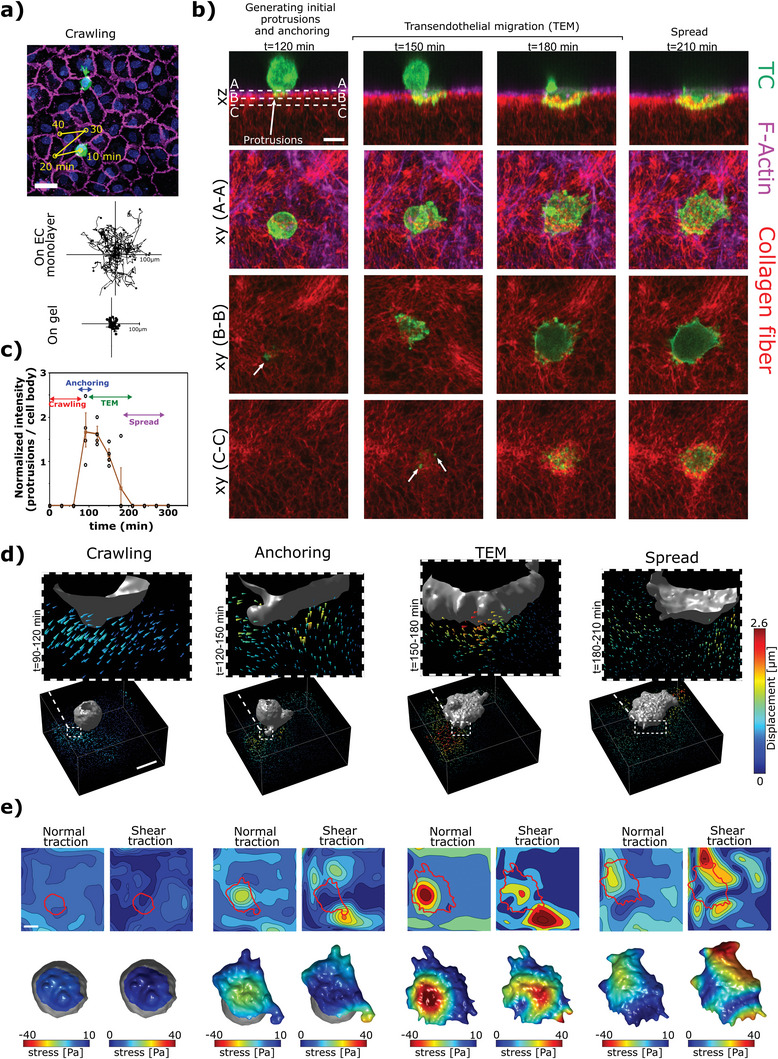
Tumor cell extravasation dynamics and transmigration forces. a) Top: the trajectory of a TC (green) crawling on top of an endothelial monolayer (magenta staining the junctions and blue the nuclei). The TC dynamically crawled until it initiated transmigration through EC junctions. Middle and bottom panels show trajectory of multiple TCs on either an endothelial monolayer or a plain collagen gel. Scale = 50 µm. b) Different stages of transmigration captured by time lapse *xyz*‐imaging of a TC (LifeAct in green) interacting with endothelial monolayer (LifeAct in magenta) and the subendothelial collagen matrix (red). TC morphology and protrusions at three *z*‐sections are represented in A–A, B–B, and C–C *xy* views. The white arrows point to initial small protrusions. Scale = 20 µm. c) Ratio of actin intensity in the protrusions compared to the actin intensity in the whole cell body at different stages of extravasation. d) Displacement fields in the vicinity of the TC at different stages of transmigration. Arrow heads indicate the displacement vectors and color scale shows the displacement magnitude. Scale = 20 µm. e) Incremental normal and shear stress fields calculated from the displacement fields of the same cell in (d). Top panels show the stress fields on the gel and the bottom panels show the projected stress fields on the areas of the TC that were in direct contact with the subendothelial ECM (indicated with solid red lines). Scale = 20 µm.

Next, we introduced TCs on top of the EC monolayer to study the complex mechanical interactions between cells and the subendothelial ECM during TC extravasation. Following their addition, the TCs settled on the EC monolayer and randomly crawled over the surface of the endothelium (**Figure** **3**a). Comparing TC trajectories on the endothelium versus on pure collagen gels indicated that, while in both cases the trajectories exhibit a random pattern, the distances travelled were significantly shorter for TCs on collagen gels versus TCs on EC monolayers, that is until the TCs arrested and adhered firmly to EC junctions on the monolayer surface (Figure 3a). In addition, the duration of TC crawling was significantly longer in the presence of the EC monolayer (Cnt vs No EC cases, Figure S14a, Supporting Information). This suggests that the endothelium is a tight barrier to TCs and that TCs must actively move on the surface of the endothelium until reaching an appropriate adhesion site for TEM. Termination of TC random motion on the endothelium was concomitant with the appearance of thin actin rich protrusions in TCs,^[^ ^18^ ^]^ which poked through EC junctional gaps into the subendothelial region (Figure 3b, Figure S15, Supporting Information). This was followed by the development of further protrusions that penetrated deeper into the subendothelial ECM. The fluorescence intensity of actin rich protrusions in TCs was ≈1.7 times higher than in the cell body (Figure 3c). Finally, after a few hours, TC morphology transformed from spherical to a flattened elliptical shape that spread under the endothelium and created large deformed regions in the subendothelial ECM (Figure 3b).

To investigate the magnitude and directionality of the forces involved during different stages, we tracked the displacements of fluorescently labelled collagen fibers by FIDVC algorithms (Figure 3d and Note S5, Figures S16–S20, Videos [Link], [Link], [Link], [Link], [Link], Supporting Information). Interestingly, during the initial stages of extravasation that involve firm adhesion of the TC and development of small TC protrusions (*t* < 120 min, Figure 3b), the displacement of collagen fibers in the vicinity of the TC was directed toward the TC and endothelium (Figure 3d). This suggests that the initial protrusions act as anchors and pull on the ECM after establishing firm adhesions. On the other hand, in later stages of TEM (120 < *t* < 180 min, Figure 3b), displacements exhibited lateral and pushing components (Figure 3d), which facilitated passage of a large proportion of the TC through the endothelial gap. Following the spreading of TC (*t* > 180 min), the magnitude of displacements dropped drastically, suggesting successful TC transmigration and shape transformation from spherical to spread. By implementing a depth‐dependent linear elastic model for the characterized subendothelial ECM (Figure 1e), the stress fields were estimated (Figure 3e). Interestingly, the maximum stress in the order of ≈40 Pa was generated during the stage at which the TC underwent dramatic morphological changes (Figure 3e) showing the direct correlation between the level of force generation and shape change.

### 2.5 Impact of Mechanical Perturbations

**Figure 4:**
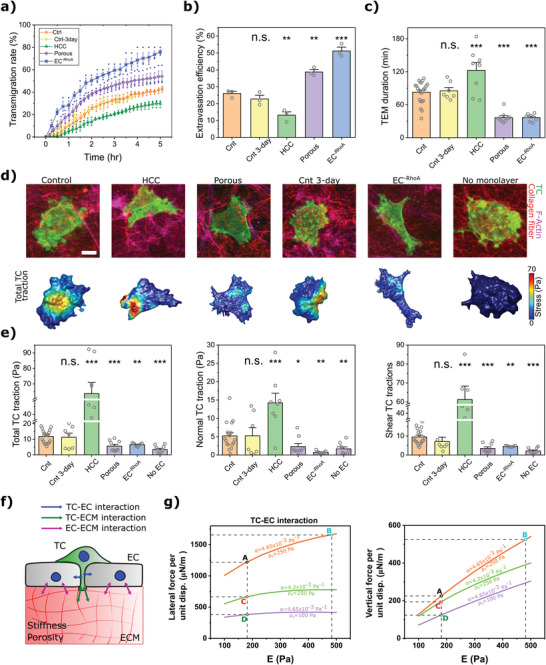
The impact of forces on TEM. a) Comparison of percentage of transmigrated TCs versus time. b) Transmigration efficiency, that is, percentage of transmigrated TCs after 3 h (For panels (a) and (b) *n* = 3 dishes and error bars represent mean ± S.E.M.). c) The duration of the transmigration for different conditions (mean ± S.E.M., *n* ≥ 7 dishes. In each dish three randomly chosen ROIs were imaged using a confocal microscope. Each data point represents the mean TEM duration for each dish). d) Confocal images showing the morphology of TCs during transmigration for different conditions (LifeAct TC and EC monolayer in green and magenta respectively, collagen in red). Lower images show incremental total tractions acting at the interface of the TC and subendothelial ECM. Scale = 10 µm. e) Average cumulative total, normal, and shear tractions applied by TCs during transmigration for different conditions (mean ± S.E.M., *n* ≥ 7 dishes. In each sample (dish) three randomly chosen ROIs were imaged using a confocal microscope. Data points represent the average of TEM tractions for each dish. For panels (a)–(c), and (e) *p < 0.05, **p < 0.01, ***p < 0.001, Student's *t*‐test). f) Schematic representation of the interacting forces between TC‐EC‐ECM during transmigration. The EC actomyosin contractility and mechanical properties of the ECM modulate ECs traction and tugging forces which can affect the level of TC generated forces required for successful TEM. g) Impact of stiffness of the substrate (*E*), its porosity (represented by *α*), and levels of ECs RhoA dependent contractility (*ρ*_0_) on forces required to open the EC monolayer (lateral force) and forces required to move the TC vertically toward ECM (vertical forces) predicted by computer simulations. The points A, B, C, and D correspond to experimental data for control, HCC, porous, and EC^−RhoA^ cases, respectively.

Having established a robust methodology for characterizing extravasation mechanics, next we evaluated the effects of perturbations in EC contractility and subendothelial ECM properties. Interestingly, the endothelium formed on HCC gels showed the strongest barrier to extravasation (49% decrease in extravasation efficiency compared to control, *p*‐value = 0.006, **Figure** **4**a,b), while TCs had the highest tendency to extravasate in RhoA depleted endothelium (2‐fold increase compared to control, *p*‐value = 0.0006, Figure 4a,b). The porous case also led to increased extravasation efficiency (1.5‐fold increase compared to control, *p*‐value = 0.003, Figure 4a,b). Moreover, extending culture duration to 3 days had no influence on the extravasation rates and efficiencies (Figure 4a,b, *p*‐value = 0.27).

The time it took TCs to transmigrate through the endothelial barrier under different conditions also showed similar trends. In the case of HCC gels, the TEM time was 1.5‐fold higher (*p*‐value = 8 × 10^−4^, Figure 4c), whereas both porous and EC^−RhoA^ cases showed a significant decrease in TEM time (56%, *p*‐value = 1.8 × 10^−7^ and 56%, *p*‐value = 1.4 × 10^−6^, respectively, Figure 4c). However, the TEM duration was not changed when the culture time of endothelium was extended (Cnt 3‐day vs control, *p*‐value = 0.76). Interestingly, the duration of the crawling or anchoring stages was not significantly different between control, Cnt 3‐day, HCC, porous, and EC^−RhoA^ conditions (Figure S14, Supporting Information). Altogether, these results suggest that the TEM stage is the rate limiting step of the extravasation.

Finally, we characterized the impact of the above perturbation on the magnitude and distribution of stresses at the TC‐ECM interface (Figure 4d,e, Figures S16a,b–S20a,b, Supporting Information). In all cases, the shear component of the stress was typically larger than the normal component. Moreover, the highest level of traction forces was observed during the “spread” stage while the lowest tractions were associated with the “anchoring” stage (Figures S16c–S20c, Supporting Information). Also, TC‐induced tractions were dramatically higher for the HCC gel case compared to control (5.4‐fold increase, *p*‐value = 2.1 × 10^−10^), while levels of stress were significantly lower for the porous and EC^−RhoA^ conditions (52% decrease, *p*‐value = 7.4e−4 and 44% decrease, *p*‐value = 0.002, respectively). Furthermore, the levels of traction forces did not change for the Cnt 3‐day condition (*p*‐value = 0.82 compared to the control). Strikingly, tractions generated by TCs when interacting with the intact collagen gel in the absence of the EC monolayer were significantly lower (71% decrease, *p*‐value = 1.0 × 10^−5^, Figure 4e).

Taken together, porous, and EC^−RhoA^ conditions led to decreased TEM duration and traction forces generated by TC, while higher levels of TC‐induced forces and longer TEM duration were observed for the HCC condition.

### 2.6 Predictive Model of Endothelial Gap Expansion and TEM

To gain mechanistic insights and to investigate the biomechanical mechanisms associated with the opening and expansion of EC junctional gaps during TEM, we extended our model of the endothelium mechanics (Note S4, Supporting Information) to include a TC passing through an opened endothelial gap (Figure S13b, Supporting Information). Indeed, we and others previously showed that TCs transmigration is initiated through penetration of small protrusions into small gaps within EC junctions^[^ ^18^ ^]^ while the successful passage of TC body requires further expansion of the gaps. During gap size expansion, TC protrusions are in physical contact with ECs, and enlarge the gap by pushing the ECs laterally (Figure 4f), while the tips of the TC protrusions interact with the ECM by applying vertical forces (Figure 4f). Using our model, we predicted the lateral and vertical forces under control, HCC, porous, and EC^−RhoA^ conditions (denoted by points A, B, C, and D in Figure 4g and Figure S21, Supporting Information). We found that higher levels of ECM stiffness (*E*) increased both lateral and vertical forces, meaning that TCs needed to apply more lateral forces on ECs to enlarge the EC gap, and higher vertical forces on the ECM to infiltrate through the monolayer (Figure 4g). Furthermore, for porous subendothelial ECM (simulated by considering a lower feedback parameter, *α*), TCs required weaker lateral and vertical forces (Figure 4g). Notably, reducing EC contractility (by decreasing *ρ*_0_, representing the EC^−RhoA^ condition) limited the level of lateral and vertical forces (Figure 4g).

## 3 Summary and Discussion

A key biomechanical barrier to the metastatic dissemination of TCs is the microvasculature of target organs. Beyond its oxygen/nutrient transport and waste collecting roles, the mechanical properties of tissue microvasculature may determine what kind of TCs (i.e., their organ of origin) are likely to extravasate and proliferate into secondary tumors.^[^ ^52^ ^]^ Circulating TCs adhere to or become trapped in the vasculature of various distant organs, actively transmigrating through the microvascular walls into the surrounding tissue and, over time, establishing metastatic tumors (known as the seed‐and‐soil paradigm). The role of chemokines and intracellular signaling pathways has been widely studied during arrest, adhesion, and TEM. For example, it has been shown that integrins are critical in the arrest of cancer cells in lung.^[^ ^11^ ^]^ On the other hand, physical, and mechanical factors, such as hemodynamic forces and capillary diameter, have also been shown to be important in the arrest, adhesion, and extravasation of TCs to distant organs.^[^ ^53^ ^]^

In order to transmigrate, TCs must disrupt endothelial integrity which is maintained by adherens junctions (AJs), as well as cytoskeletal structural elements of ECs.^[^ ^54^ ^]^ AJs are mechanosensitive cadherin‐based intercellular adhesion sites that interact with the actin cytoskeleton.^[^ ^55^ ^]^ Similarly, TC protrusion dynamics and both TC and EC shape changes occurring during TEM rely on actin cytoskeletal rearrangements and actomyosin contractility, which are modulated by intracellular signaling pathways. For instance, Rho GTPase signaling networks regulate TEM, adjust TC–EC interactions, and are involved in the disruption of endothelial barrier function.^[^ ^12^ ^]^ Despite their critical role, our knowledge of the actomyosin‐dependent forces involved during TEM is scarce, partly due to the lack of appropriate assays to interrogate them. Furthermore, the subendothelial ECM is another crucial element which is mostly overlooked in cancer extravasation studies, despite its prominent role in regulating EC–EC and TC–ECM interactions.

Here, we developed a robust TC extravasation assay (Figure 1) with the key advantage of measuring forces generated by the endothelial monolayer (Figure 2), as well as those associated with transmigration of TCs through endothelium (Figure 3). Furthermore, the subendothelial ECM stiffness and porosity and/or the intracellular signaling in ECs could be readily perturbed, thus enabling the investigation of the molecular/biomechanical determinants of TEM. Our experimental results, in combination with computer simulation data revealed that the compliance or porosity of the subendothelial ECM regulates EC intercellular contractile forces generated by the actomyosin complex and mediated through RhoA/MLC2 activity. Strikingly, we found that the mechanical properties of ECM also impact TEM, either directly through TC–ECM interactions or indirectly through regulation of TC–EC intercellular forces.

Noteworthingly, in this study, we varied the collagen concentration to tune the stiffness of the subendothelial matrix. However, increased collagen concentration may also affect a wide range of biophysical factors including fiber dimensions (length^[^ ^56^ ^]^ and diameter^[^ ^40^, ^57^ ^]^), fiber density,^[^ ^58^ ^]^ pore size,^[^ ^57^ ^]^ and interconnectivity between fibrils.^[^ ^59^ ^]^ Here, our SEM images showed significantly smaller pore size for the HCC case which is in agreement with previous studies.^[^ ^60^ ^]^ This change in the pore size can lead to changes in cell–ECM interactions through several factors (such as changes in available functional groups or anchorage points for the cells), which in turn can result in modulation of cellular behavior (for example changes in size and frequency of focal adhesions,^[^ ^61^ ^]^ migratory behavior,^[^ ^62^, ^63^ ^]^ polarity,^[^ ^61^ ^]^ F‐actin expression level,^[^ ^64^, ^65^ ^]^ etc.). In the current study, our emphasis was on the calculation of forces exerted by ECs or TCs during TEM and establishing a correlation between these forces and the transmigration rate or duration, when subendothelial matrices with different structural properties were used. Therefore, in our study we investigated the combined effects of such biophysical determinants by using “high collagen content (HCC)”. However, dissecting the impact of each individual biophysical parameter would be an interesting goal for future follow‐on studies.

Altogether, our data suggest that extravasation efficiency correlates with the level of forces generated by either TCs or ECs during TEM. Particularly, the force‐generating ability of ECs depends on RhoA activity and thus downregulation of RhoA in ECs led to increased extravasation rates and decreased TEM durations. Consequently, we propose that the level of traction or tugging forces that are generated by ECs and tuned by mechanical properties of subendothelial ECM, can be considered as a novel index to evaluate the extravasation potential. Endothelial permeability has been suggested as an indicator of TC extravasation capability.^[^ ^66^ ^]^ However, a perfect correlation between extravasation rate and the endothelial permeability cannot be found in many cases.^[^ ^1^ ^]^ For example, it has been suggested that culturing ECs on stiffer substrate may lead to increased endothelial permeability^[^ ^67^ ^]^ while we and others found that cancer extravasation decreases when stiffer subendothelial ECM is used.^[^ ^68^ ^]^ Therefore, since different time and length scales as well as mechanisms are involved in the permeation of solutes across the endothelium compared to the transmigration of whole TCs, we suggest that the integrity and the magnitude of forces associated with the endothelium (Figure 2) are a better predictor of cancer cell extravasation potential.

It is worth noting that in the current study, we used a 2D in vitro approach to investigate the mechanical interaction between EC, TC, and ECM and therefore, care must be taken when generalizing the results to 3D in vitro vascular systems or in vivo. Indeed, due to complexities in quantifying / altering the mechanical properties of tissues, the in vivo study of such interactions during TC transmigration is extremely difficult, if not impossible. Building on our refined techniques, we envisage that 3D microvasculature networks^[^ ^69^ ^]^ formed within microfluidic platforms^[^ ^70^ ^]^ will enable detailed investigation of transmigration mechanics in more physiologically relevant conditions.

In the context of organ specificity of metastases and the seed‐and‐soil paradigm, a fundamental understanding of the mechanisms that underlie TC transmigration across different types of microvasculature with different biomechanical features may inform new methods to inhibit TEM. Importantly, these advances may lead to the development of novel therapeutic strategies to reduce metastasis and improve cancer patient survival rates.

## 4 Experimental Section

### Cell Culture

HUVECs (Lonza, passages 8–10) transfected to express LifeAct‐mCherry were passaged and cultured in EGM‐2MV media (Lonza). MDA‐MB‐231 (Lonza) expressing LifeAct‐GFP, were cultured in DMEM media supplemented with 10% FBS and 1% penicillin‐streptomycin. Cells were initially expanded and cryopreserved to establish a consistent stock for all experiments. For an individual experiment, ECs were plated onto collagen‐coated flasks and grown to confluency before being introduced into the assay.

### Collagen Gel Preparation

Rat‐tail type I collagen (BD Biosciences) was diluted in HEPES buffer (5% v/v) and 10X PBS with phenol red (10% v/v) and was brought to physiological pH (pH = 7.4) with 0.5 n NaOH. Sterile water was added at the end to adjust the volume. The final concentration of collagen, for the control and porous gels, was 2.5 mg mL^−1^ while it was 6 mg mL^−1^ for the HCC gel. All materials were kept on ice during the procedure. Then, 40–45 µL of collagen mixture was spread onto glass bottom dishes (MatTek Life Science) of 10 mm diameter, previously coated with 5% glutaraldehyde to enhance the gel‐glass attachment. For the control and HCC conditions, the gel was incubated at 37 °C for 30 min, while the porous case was incubated at 20 °C for 60 min. Following collagen gelation PBS was added to the dishes.

### Endothelium Formation and Staining

Prior to seeding ECs on gels, the collagen fibers were stained with 50 µm 5(6)‐Carboxytetramethylrhodamine succinimidyl ester (5‐TAMRA SE, Invitrogen, Carlsbad, USA) at room temperature for 1 h and rinsed 5 times with PBS. To form the endothelial monolayer, PBS was replaced by 2 mL of EGM‐2MV containing 350 000 HUVECs. EC density was optimized by culturing EC with different initial densities (Note S6, Figure S22, Supporting Information). Also, the impact of EC density on transmigration rate was evaluated by introducing 700 000 ECs to the dish (Figure S23, Supporting Information). The dish was kept in the incubator (37 °C, 5% CO_2_) for 48 h (except for the Cnt 3‐day condition for which the dish was incubated for 3 days) with media change every 24 h. To visualize cell nuclei and EC junctions, the dish was incubated for 5 min with Hoechst (New England Biolabs) and anti‐human CD31 Alexa 647 (BD BioScience), respectively. The fluorescently stained EC monolayer was then imaged using a confocal microscope (Olympus, FV1000, Japan) and 60× objective. For characterization of collagen properties, the ECs were washed off using Triton X treatments. Z‐stack confocal images of collagen fibers were acquired using 60× objective. The acquired images were binarized using ImageJ and the structural quantifications for different conditions were caried out using the method described in supplementary note 2.

### Extravasation Assay

Following formation of tight endothelium, 25000 MDA‐MB‐231 cells were added to the dish, prior to acquiring images with an appropriate microscope. An epifluorescent microscope (Leica, DMi8, Germany) was used to assess extravasation rate and efficiency, while a confocal microscope (Olympus, FV1000, Japan) was employed to evaluate transmigration duration and traction forces. Epifluorescent microscope images were taken every 5–10 min over 6 h using a 10× objective. The confocal z‐stacks (50‐100 *z*‐planes, 0.5 µm apart) were captured using a 60× objective every 15–30 min over 4–8 h.

To quantify extravasation rates, 3 samples (dishes) were used for each scenario, and for each dish, 3 randomly selected ROIs were imaged using a 10× objective. Transmigration rate was determined by dividing the number of transmigrated cells by the total number of cells in the ROI and these values were then averaged to find the transmigration rate for each dish at each time step (data points in Figure 4a). The rate of extravasation after 3 h was plotted as extravasation efficiency (Figure 4b) and hence, data points represent the mean transmigration rate in each dish after 3 h.

### Atomic Force Microscopy

AFM characterization of the mechanical properties of gels has been described in detail previously.^[^ ^71^, ^72^ ^]^ Briefly, AFM force‐distance measurements were acquired using a JPK Nanowizard Cellhesion 200 (JPK Instrument, Germany). Spring constants of the tipless cantilevers (MLCT‐O10, Bruker; nominal spring constant of 0.07 N m^−1^) were determined using the thermal noise method of the AFM software (JPK SPM, JPK Instrument). Cantilevers were modified by gluing 25 µm diameter glass microspheres (Cospheric, USA) to the tip of the cantilever via UV curing glue (ultraviolet curing, Loctite). The stage was carefully moved to position the cantilever tip above the middle of the sample prior to approaching the gel surface. An approach speed of 5 µm s^−1^ and set forces up to 30 nN was used to attain the force‐distance curves which were used to quantify the elastic modulus of the gels (Note S2, Supporting Information). To quantify the elastic modulus of the collagen gels, measurements were carried out on a minimum of 20 randomly selected points in each dish and the average of the values were reported as the sample's elastic modulus (represented by each data point in Figure 1g). To quantify the elastic modulus of control, HCC, and porous gels, eight samples were used for each condition.

### Permeability Measurement

Fluorescent dextran tracer (70 kDa FITC‐dextran; 10 µg mL^−1^ in EGM‐2MV; Life Technologies) was added to the dish to assess the permeability of the EC monolayer. Dextran, endothelium, and the underlying matrix were imaged with a 20× objective for 40 min every 1 min using confocal microscope (*z*‐line sections covering 100 µm above and below monolayer). The permeability was calculated using the following equation:

(1) $$P=\frac{1}{t_{2}-t_{1}}\;\frac{I_{\mathrm{gel}}^{t_{2}}-I_{\mathrm{gel}}^{t_{1}}}{\left[\left(I_{\mathrm{top}}^{t_{2}}-I_{\mathrm{gel}}^{t_{2}}\right)+\left(I_{\mathrm{top}}^{t_{1}}-I_{\mathrm{gel}}^{t_{1}}\right)\right]/2}d$$

in which *I*_gel_ and *I*_top_ are the average intensity of the tracer in the gel and above the EC monolayer, respectively. Superscripts *t*_1_ and *t*_2_ represent initial and final time points and *d* is depth of the gel.

### Force Quantification

To quantify the forces (Figure 2e,g), time‐lapse confocal stacks of the fluorescently labeled collagen fibers were acquired, and the displacements were obtained using the FIDVC algorithm.^[^ ^49^ ^]^ For force quantification of EC monolayer, incremental displacements were obtained by comparing two consecutive images (acquired at *t* and *t* + Δ*t*), while the cumulative displacements at each time (*t*) was calculated by comparing the corresponding image with the stress‐free reference state (*t*_ref_) obtained after cytochalasin‐d treatment on the monolayer. To quantify forces generated by TC during extravasation (Figure 3e and Figure 4d,e), the image acquired at the beginning of the anchoring stage was used as the reference state. A commercial finite element (FE) software (ABAQUS, Dassault Systèmes) was used to simulate the mechanical interactions between the cells and the substrate.^[^ ^73^ ^]^ Briefly, the subendothelial ECM was modelled as a cuboid. A quadratic tetrahedral 10‐node element (C3D10M) with second‐order accuracy was employed to mesh the domain after mesh sensitivity analysis. A linear elastic constitutive model was used to describe the mechanical behavior of the gel considering the Poisson's ratio of 0.2^[^ ^74^ ^]^ and elastic modulus as a function of depth (Note S2, Supporting Information). A large deformation formulation was taken into account to consider the effect of geometric nonlinearity.^[^ ^75^ ^]^ To apply boundary conditions, nodes located at the bottom surface were constrained in three directions, while no restrictions were applied to the nodes at the side of the domain.

The displacements field obtained from the FIDVC code were applied to the nodes located at the top surface of the gel. A dynamic implicit solver was then used to compute traction stress fields in the gel. The components of stress tensors and deformation vectors were extracted from “.odb” output files using a custom‐written Python script. An image processing toolbox (MATLAB, MathWorks) was employed, in combination with ImageJ, to smoothen and display stress/displacement fields on the cancer cells or the subendothelial ECM. The incremental force generated by EC monolayer or TC between every two consecutive time steps (*t* and *t* + Δ*t*) was calculated by integrating the stress over the area located beneath the EC monolayer or TC, respectively. To find the cumulative force at time *t_i_* all incremental forces between the time *t_i_* and the reference state *t_ref_* were added together. Finally, to calculate the average cumulative stress at *t_i_*, the estimated cumulative force was divided by the area below the EC monolayer or TC.

In Figure 2g, for each condition 5 samples (dishes) were used and in each sample 3 ROIs were selected randomly and imaged to find the displacement field below the EC monolayer and calculate traction forces over time. At each ROI, mean tractions were calculated over the domain for each time step, then averaged over time, and finally the mean value for the three ROIs was calculated and presented by each data point in the figure.

In Figure 2h, similarly for each condition there were five samples, and three ROIs were chosen randomly per dish. Then, the tugging forces were calculated for each cell at each time step. These values were averaged over time to find the mean tugging force for each cell, then, the average tugging force for all the cells in each ROI were calculated and finally the mean tugging force for each sample was computed by averaging the mean values of ROIs. Data point in Figure 2h represents the mean tugging force per sample.

To measure TEM duration and to quantify TC tractions ten samples (dishes) were used for each condition. In each dish, three randomly selected ROIs that contained a TC were imaged using a 60× objective. TEM duration or TC tractions were calculated only for those ROIs in which the TC managed to transmigrate within the experimental time frame. Each data point in Figure 4c,e represents the mean value for each sample. Also, experiments carried out for each condition had their own control. Therefore the “Cnt” case has a higher number of data points compared to other conditions.

To verify the accuracy of the force quantification methodology, a known force was experimentally applied to the surface of the gel and followed the steps mentioned above to reconstruct the force and then compared the applied and reconstructed forces (Note S7, Figure S24, Supporting Information). To achieve this, the AFM head was placed on a confocal microscope (Olympus, IX81, Japan) and *z*‐stack images of collagen fibers were acquired before and after indentation of the collagen top surface. This methodology may lead to errorsup to 20% in the estimation of the reconstructed forces (Note S7, Figure S25, Supporting Information).

### siRNA Transfection

ECs were seeded on the control gel to form a confluent monolayer. Following formation of the EC monolayer at day 2, the media in the dish was replaced with 2 mL serum free media containing delivery reagent (Fisher Scientifics Ltd) and siRNAs (RhoA siRNA from Dharmacon‐US) at a final concentration of 50 nm. Transfection medium was replaced after 16 h of transfection with regular culture medium to avoid toxicity. The efficiency of the transfection was examined by conducting Western blots (all materials were obtained from Bio‐Rad Laboratories Ltd unless otherwise stated) on the cells treated with siRNA. To separate the proteins by their mass, the whole‐protein content isolated from lysed cells was run through sodium dodecyl sulfate–polyacrylamide gel electrophoresis (SDS‐PAGE). The separated proteins transferred to the nitrocellulose membrane were subsequently stained with primary antibodies (RhoA, Insight Biotechnology, US and *β*‐actin, Sigma‐Aldrich, US). Following 24 h the secondary antibody (Thermo Fisher Scientific, US) was applied, followed by exposure to revealing reagent (Bio‐Rad, US) and imaging by Amersham Imager 680.

### Sample Preparation for SEM

Collagen samples were prepared in glass bottom (MatTek Corp, US) dishes and were fixed using 5% paraformaldehyde in 0.1 m PBS for 30 min and washed thoroughly with deionized water three times. The samples were dehydrated by 30%, 50%, 70%, 90%, and 100% (×3) graded ethanol, each for 15 min. Next, specimens were critical point dried using 100% hexamethyldisilane (HMDS). Finally, the glass part of the dish was detached from the dish by a gentle pressure of tweezers and mounted on SEM stud. The collagen containing region of the glass was sputter coated with carbon and gold and its periphery was then coated with silver.

### Calculation of Fiber Thickness and Pore Size from SEM Images

Fiber diameters were calculated using the straight‐line tool in ImageJ. At least 200 measurements were performed for each ROI and three images were analyzed per dish. To evaluate pore size (Figure S2a, Supporting Information), images were binarized using “Trainable Weka Segmentation” plugin in ImageJ (Figure S2b,c, Supporting Information). Then, the area of each pore was calculated using “Analyze‐Particle” tool in ImageJ (Figure S2d,e, Supporting Information). Finally, a customized code in MATLAB was employed to find the diameter of the largest circle that could be inscribed in each pore (Figure S2f, Supporting Information). Pore size was calculated for the three images captured per specimen and average values for each dish were represented as data points in Figure 1f.

### Statistical Analysis

At least four samples from three independent experimental repeats were analyzed for quantification and statistical comparison. Quantification, statistical analysis, and plotting were performed in MATLAB (MathWorks) or Origin (OriginLab). Data are presented as mean ± S.E.M., and *p*‐values < 0.05 were considered statistically significant (*p < 0.05, **p < 0.01 and ***p < 0.001).

## Conflict of Interest

The authors declare no conflict of interest.

## Author Contributions

Y.J., F.C., R.K., and E.M. conceived the study and designed the experiments and simulations. E.M. performed the experiments with the help of Y.J., A.A., S.L., S.B., M.C., L.C., R.L., and M.J. Y.J. and E.M. conducted simulations with help from A.M., F.S., and V.S. Y.J. and E.M. processed and analyzed the experimental data with the help of S.S., B.S., S.S., and G.K.S. Y.J. and E.M. wrote the manuscript and F.C., G.K.S., and R.K. edited the manuscript. R.K. and E.M. supervised the study.

## Supporting information

Supporting Information

Supplemental Video 1

Supplemental Video 2

Supplemental Video 3

Supplemental Video 4

Supplemental Video 5

Supplemental Video 6

## Data Availability

The data that support the findings of this study are available from the corresponding author upon reasonable request.
