# Supplementary material for: Endothelium and Subendothelial Matrix Mechanics Modulate Cancer Cell Transendothelial Migration
Source: Adv Sci (Weinh). 2023 Apr 13;10(16):2206554. doi: 10.1002/advs.202206554 (PMC10238207; doi:10.1002/advs.202206554)
Supplement: Supplementary file 1 — Supporting Information [file ADVS-10-2206554-s006.pdf]

Supplementary note 1- Pore size and fiber thickness of collagen matrix

To accurately quantify the fiber thickness and pore size we imaged the dehydrated samples (using graded ethanol) and employed a scanning electron microscope (Zeiss-Gemini, Germany). Collagen is the main contributor to the stiffness and strength of soft connective tissues, such as the cornea, blood vessels, ligament and tendons[1], in which the uniaxial alignment, high collagen density, hierarchical fibril architecture, and degree of crosslinking enables these tissues to resist high tensile loads[2]. However, collagen hydrogels represent relatively poor mechanical properties in the absence of covalent cross-linking[3] and increasing collagen content does not seem to improve these properties tremendously. Therefore, applying some modifications, such as using crosslinkers, seems to be more physiologically relevant than elevating collagen concentration. Furthermore, in contrast to some other hydrogels present in native ECM, collagen hydrogels do not have any growth factor (GF)-specific binding sites and often cannot sequester physiological amounts of the protein[3] and again employing chemical cross-linkers would provide a physiologically relevant strategy to improve the hydrogel GF-binding properties.

Such limitations have triggered many studies on collagen gels to provide a more physiologically relevant platform disassociating the effects of hydrogel stiffness from other factors. For instance, to de-couple matrix stiffness from matrix density and structure in collagen gels, Mason et al.[4] used non-enzymatic glycation of the collagen in solution, prior to polymerization to increase the compressive modulus of the hydrogel without significant changes to its architecture. They demonstrated that matrix stiffness alone can promote spreading of endothelial cells within the 3D matrix and consequently, number and length of the angiogenic sprouts enhanced. Additionally, carbodiimides[5], polyethylene glycol[6], glutaraldehyde[7], isocyanate[8], glycation with glucose[9] or ribose[4], and genipin[10] have been used as chemical cross-linkers to improve mechanical properties of collagen hydrogel. An interesting avenue to explore in future studies would be the use of crosslinkers to tune mechanical properties of the matrix without altering its structural architecture to provide a better picture of the mechanical behaviour of EC / TC during transmigration.

Gelation temperature has a significant effect on polymerization kinetics that will largely determine the mechanical properties of collagen hydrogels. Fibrillogenesis occurs faster at higher temperatures due to accelerated nucleation and lateral aggregation of collagen molecules[3]. Shannon et al.[11] showed that for 3 mg/ml collagen I concentration, increasing gelation temperature from 18 °C to 37 °C leads to a reduction in polymerization time. Similar observations were reported by other researchers[12,13]. Furthermore, our SEM images show that reducing the temperature results in the formation of thicker fibers. Other researchers also observed a similar pattern using SEM[12,14,15], confocal microscopy[16], confocal reflectance microscopy[17], multiphoton microscopy[15], and second harmonic generation microscopy[18]. It has also been shown that fibers polymerized at higher temperatures are shorter, randomly aligned, and less bundled[19]. Lower temperatures are thought to limit nucleation of new fibers via decreasing entropy, which promotes thickening and elongation of already existing fibers, and forms networks, which are often more heterogeneous[3,20]. Additionally, in agreement with previous studies[15–17,19,21], our SEM images show that pore size increases with decreasing gelation temperatures for the same level of collagen content (**Fig 1f, S1, S2**). Additionally, a higher permeability for the porous gel would provide additional evidence indicating its larger pore size **(Fig S3)**.

However, regarding mechanical properties, there is no agreement among researchers; some studies have shown that higher gelation temperature elevates the stiffness[21], while other researchers have shown that greater compressive or shear moduli are produced in gels at lower temperatures. Achilli and Mantovani[22] reported that for pH=7, increasing the temperature increased stiffness, whereas a

reverse pattern was observed for pH=10. These discrepancies are thought to depend on pH and collagen concentration, and possibly on the mechanical testing setup[3].

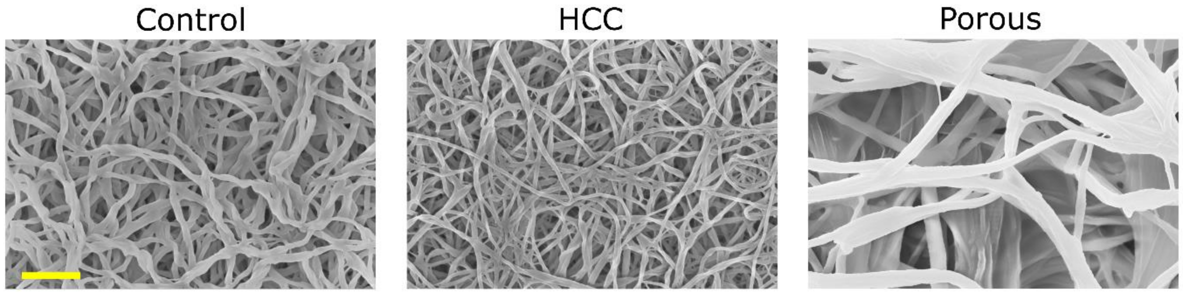

Fig S1) Scanning electron microscopy image of the control, high collagen content, and porous gels (Scale = 1 um)

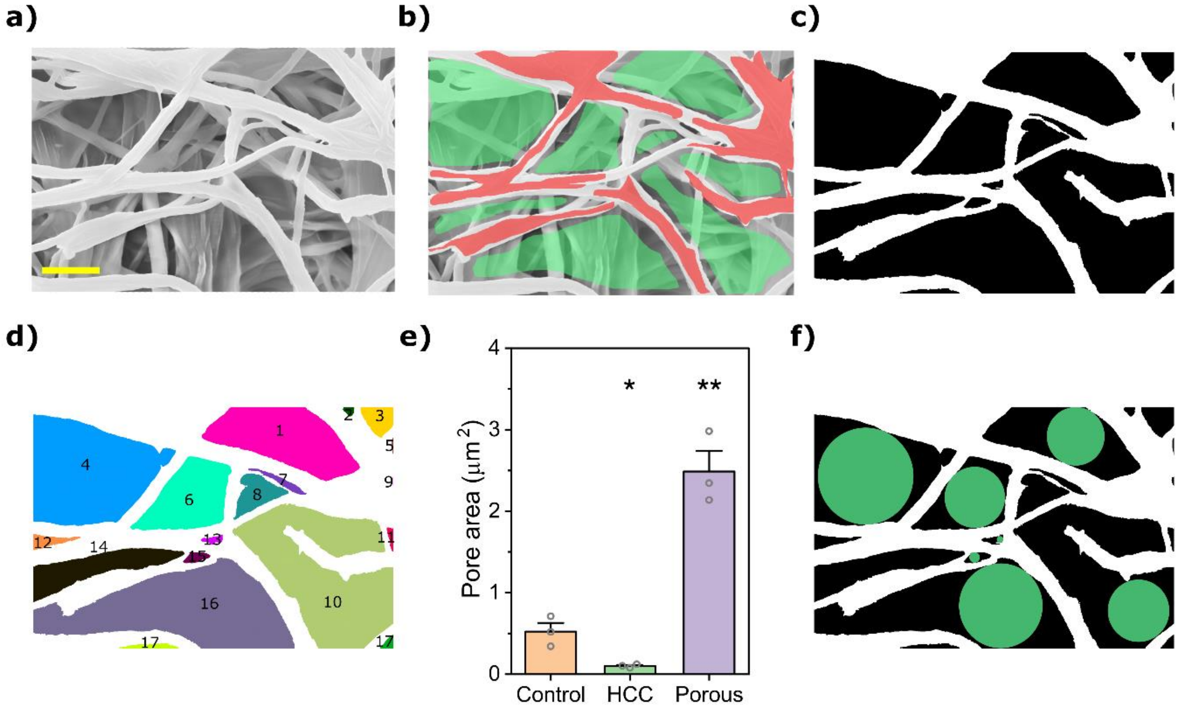

Fig S2) Quantification of pore size and pore area for hydrogels. **a)** SEM image from the sample. **b)** binarisation of the image using the "Trainable Weka Segmentation" tool in ImageJ. Only fibers located on top surface (red) were defined as region 1 while corresponding pores (green) were selected as region 0. **c)** Binarized image. **d)** Pores were labelled, and their areas were calculated using the "Analyze Particle" tool in ImageJ. **e)** The average area of pores (mean ± s.e.m., n=3 samples, in each sample, pore size evaluated at 3 randomly selected regions; data points represent the average pore area calculated for each dish; *p < 0.05, **p<0.01, ***p<0.001, Student's t-test). **f)** The biggest circle that could be inscribed in each pore (green circles) was found using MATLAB. Scale =1 µm.

Device Fabrication for permeability measurement

Acrylic moulds were fabricated by cutting (Epilog laser cutter) our designs through 1 mm thickness acrylic sheets. Next, the laser-cut acrylic geometries were bonded to larger acrylic bases using acrylic cement (Weld On 16 Clear, Multibond Solutions). These moulds were then taped to the bottom of Petri dishes to fabricate the final Polydimethylsiloxane (PDMS) devices. PDMS and crosslinker (10:1; Sylgard-184; Dow Corning) were mixed, degassed, and poured over the mould up to a height of 4 mm. After curing for 2 hours at 90° C, the PDMS was cut and peeled from the mould. Ports at the ends of the channel were punched out using 2 mm and 4 mm biopsy punches (Miltex). The device was cleaned with tape to remove debris. Then the bottom of the device and a glass coverslip were treated with plasma for 60 s and pressed firmly together to form an irreversible bond. To restore hydrophobicity of surfaces and create irreversible bonding, the final devices were placed inside an 80°C oven overnight. Next, the collagen solution was injected to the collagen channel (length = 25 mm, width = 3 mm, height = 1 mm) from the smaller port, followed by hydrogel incubation at 37°C or 20°C. Finally, to measure the permeability, 50 µL dye was added to the larger port and the devices were imaged every 15 min.

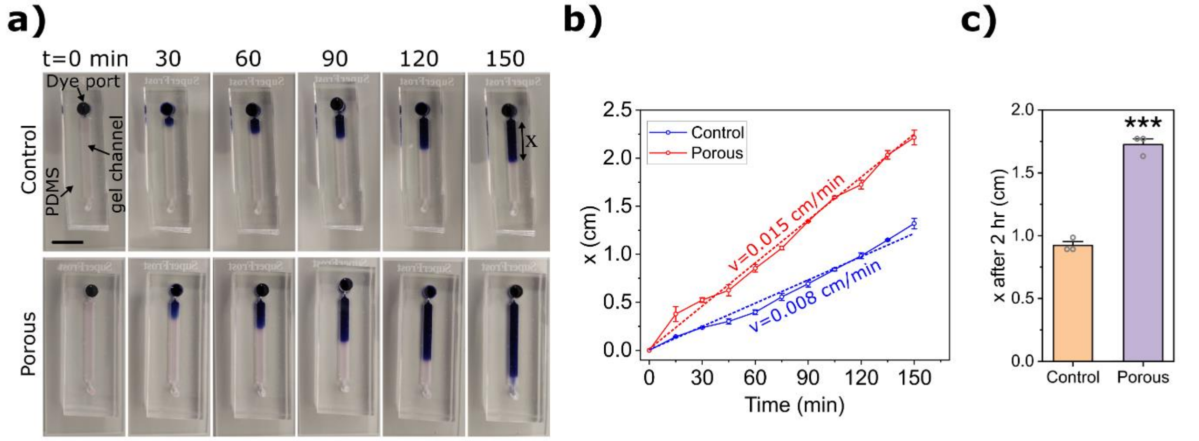

Fig S3) Quantification of the permeability of a dye in control and porous gels. **a)** Time lapse images showing the penetration of the blue dye along a gel channel made in PDMS. Scale = 1 cm. **b)** Distance travelled by the dye (x) vs time. **c)** Comparison on penetration length after 2 hr for control and porous gels (Data points represent the data obtained from each experiment, n=3 independent experiments, mean ± s.e.m.; *p < 0.05, **p<0.01, ***p<0.001, Student's t-test).

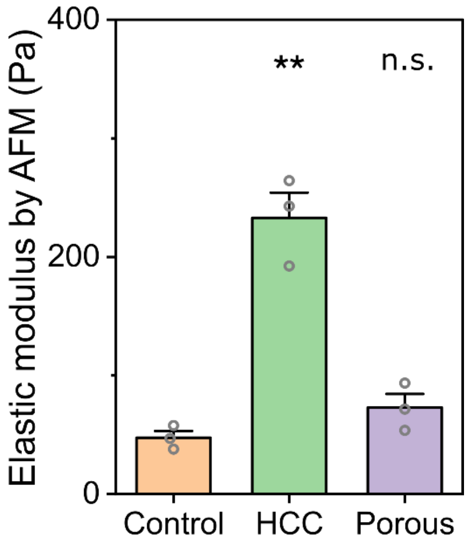

Fig S4) Elastic modulus the collagen gels measured by atomic force microscopy indentations on the top surface of the gels. Collagen gels were prepared in a glass bottom dish and were incubated in 37 °C (control and HCC) or 20 °C (Porous) for two days, then, elastic modulus was measured using AFM without introduction of any cells to the dish (mean ± s.e.m., n =3 dishes. In each dish at least 40 measurements were carried out. Data points represent average elastic modulus of each gel; *p < 0.05, **p<0.01, and ***p<0.001, Student's t-test).

Supplementary note 2- Quantification of stiffness of the subendothelial matrix

In this note, we explain how the Elastic modulus of the subendothelial collagen matrix was calculated as a function of depth. The stiffness of the collagen gel was assumed to be uniform before seeding of the endothelial cells (ECs) on top. However, once formed, the endothelial monolayer remodeled the underlying collagen gel by applying shear and compressive forces. This led to a denser and stiffer layer of collagen fibers at the top of the subendothelial matrix. The next step was to characterize the stiffness of collagen at increasing depths through the gel. To achieve this, we used the following information:

(1) $E_{initial}$: the stiffness of the collagen gel was measured before adding the ECs, using atomic force microscopy (AFM, **Fig S5**).

(2) $E_{apparent}$: the apparent stiffness of the collagen was quantified using AFM after decellularization of the EC monolayer using Triton X. After washing away the monolayer of ECs, the collagen gel swelled and its thickness increased, but it remained thinner than its initial thickness before any EC remodeling had occurred. Therefore, the gel was still non-uniform in depth, and we expected it to be stiffer near its top surface. Thus, we used the word "apparent" to emphasize that the gel does not have a single stiffness value under this condition. $E_{apparent}$ represents an average stiffness of the layers near the top surface and since E decreases with increasing depth, $E_{apparent}$ should be lower than the Elastic modulus at the top surface of the gel (E_{top}).

(3) confocal images from collagen fibers before and after washing off the EC monolayer. This enabled us to quantify "mesh size" (ζ)[23] of the gel at each z-stack plane of focus. The mesh size is an index that represents the average distance between collagen fibers.

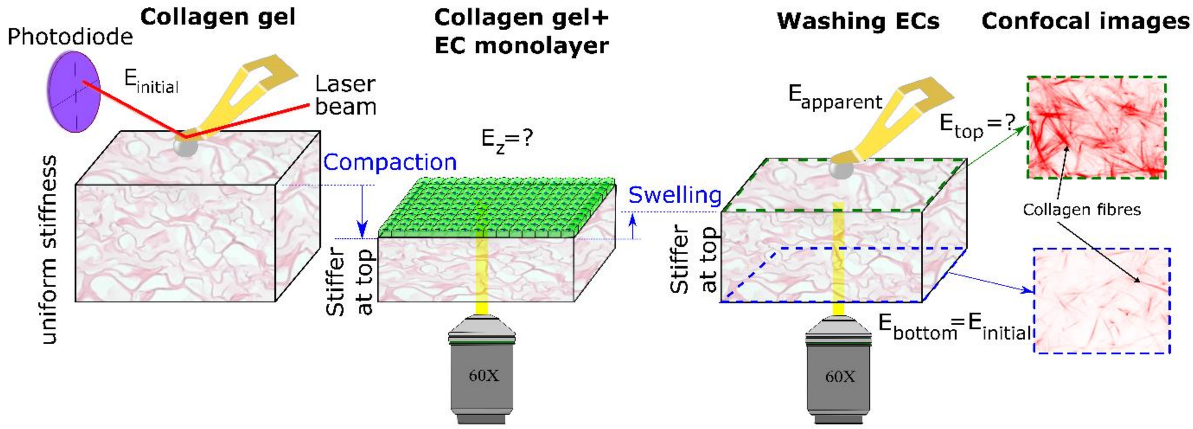

Fig S5. Quantification of the Elastic modulus of the subendothelial matrix as a function of depth.

From (3), we can calculate the mesh size at each z-plane of focus. We then needed to devise a method to correlate the Young's modulus with the mesh size to be able to estimate the Young's modulus at each plane of focus. As suggested in previous studies[23], the Young's modulus is inversely proportional to a power of mesh size:

$$E = A\zeta^{-n} \tag{S1}$$

in which *A* and *n* are two constants. To find these two parameters, we needed to know the values of the Young's modulus and the mesh size at two focus planes, such as the top and bottom of the gel. Since the bottom is far away from the EC monolayer, it would not be affected by the EC monolayer and the Young's modulus would remain equal to the initial value (E_{bottom}=$E_{initial}$). However, determining

E_{top} is not quite as straightforward and requires trial and error. In the following section, we provide a step-by-step procedure to calculate the Young's modulus for the porous gel.

Determining mesh size at increasing depths

As mentioned above, mesh size is an index representing the void size of the hydrogel. To find the mesh size (ζ), we used the protocol proposed by Yang et al.[23]. Briefly, z-stack confocal images at each plane of focus were binarized after being segmented using a thresholding algorithm in ImageJ. Then, the horizontal and vertical distances between collagen fibers were measured using a MATLAB code. The mesh size can be obtained from the exponential fit to the void size histogram (**Fig S6**). The horizontal and vertical void sizes show similar distributions. This provides further evidence that the collagen gel can be considered as an isotropic material at each focal plane.

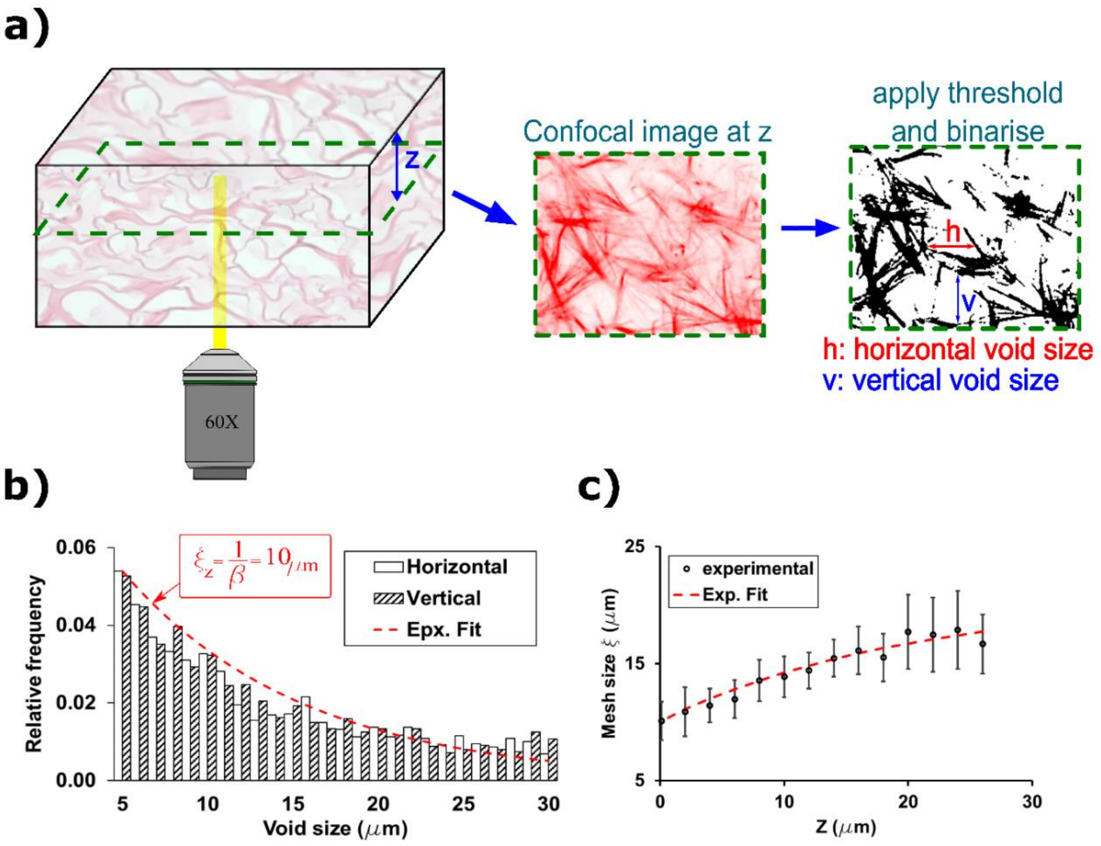

Fig S6. Calculating mesh size from confocal images. **a)** At each plane of focus, the horizontal and vertical void size between collagen fibers was measured after binarization of the image **b)** The mesh size at each focal plane was calculated from the exponential fit to the void size distribution ($relative\ frequency = \alpha\ .exp\ (-\beta.(Void\ size))$). **c)** By repeating this procedure, the mesh size can be calculated at increasing depths through the gel (Data are mean ± s.e.m., n= 8 samples).

Finally, using an exponential fit, the mesh size can be written as a function of depth. For the porous gel:

$$\zeta = 10.086 + 10.5 * (1 - e^{-0.05.z}) \quad \text{(S2)}$$

Finding the Young's modulus at the top and the exponent *n*

For the porous gel the initial and apparent Young's moduli were measured using AFM ($E_{initial}$=E_{bottom}=69 Pa, $E_{apparent}$ = 100 Pa, **Fig S7a**). Additionally, the mesh sizes were calculated in the previous step at all

focal planes, in particular, at the top and bottom of the gel (ζ_{top}=10, ζ_{bottom}=16.7). Our next aim was to estimate the Young's modulus at the top of the gel (E_{top}) by trial and error. To achieve this, we carried out the following steps:

1) We guesstimated the magnitude of E_{top}. Knowing the value of $E_{apparent}$ helped us to make an accurate estimate ($E_{top} \geq E_{apparent}$). **Fig S7a** indicates three typical guesses for E_{top} (100, 135, and 190 Pa).

2) Calculate the exponent *n* (from Eq. S1) for each guesstimate value. Since the Young's moduli and mesh sizes are known at the top and bottom of the gel, *n* can be found using the following equation for each case:

$$n = -\frac{\ln E_{top} - \ln E_{bottom}}{\ln \zeta_{top} - \ln \zeta_{bottom}} \tag{S3}$$

Using Eq. S3, the exponent *n* was found to be 1.5, 1.0, and 0.6 for E_{top}=190, 135, and 100 Pa, respectively.

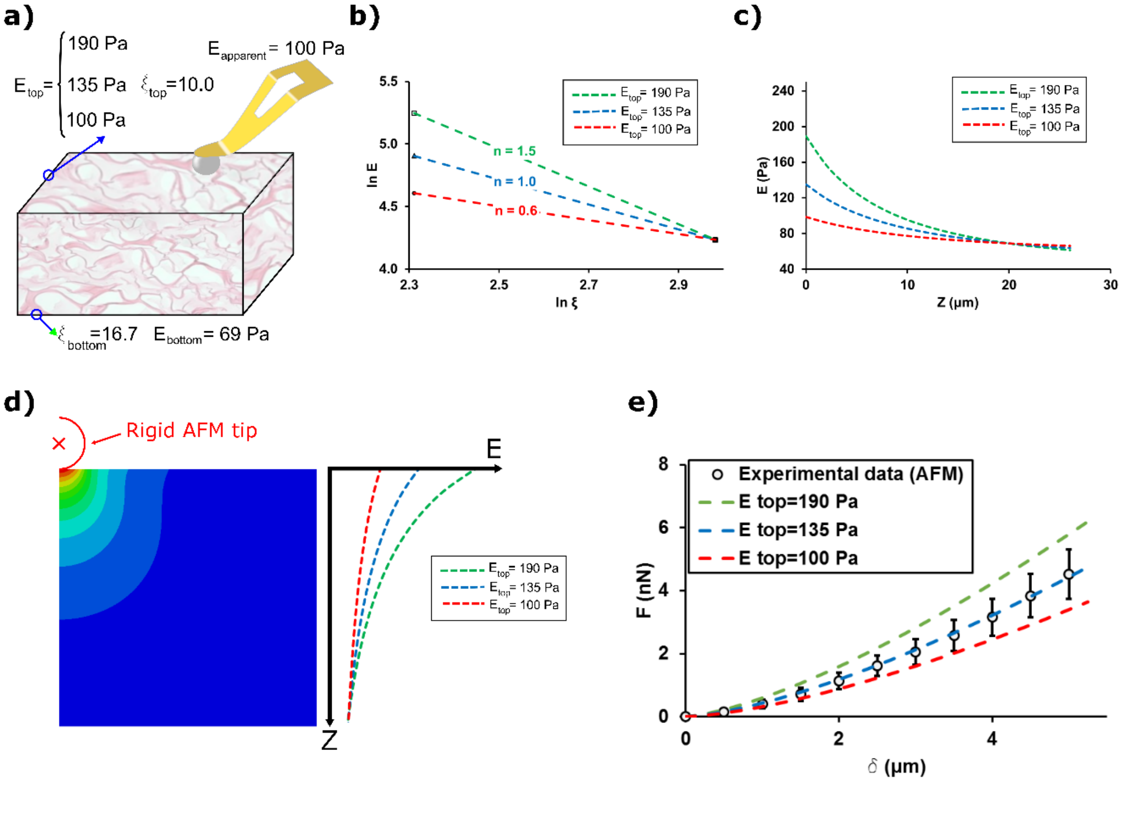

Fig S7. Determination of the Young's modulus at top and material parameters (the exponent n) **a)** The Young's modulus at the top of the gel is quantified by trial and error. Three typical guesses are shown for E_{top}. **b)** Determination of the exponent *n* for each guess. **c)** Finding variation in the Young's modulus with increasing depth for each guess. **D)** Finite element analysis (FEA) of the atomic force microscopy (AFM) experiment, assuming the depth-dependent Young's modulus. **e)** Comparison of the force-indentation curves obtained from FEA simulations and AFM experiments (mean ±s.e.m, n= 3 samples).

3) Combining Eq. S1, S2, and the value found for *n*, the Young's modulus can be determined as a function of depth (z) for each guess (**Fig S7c**):

$$E = 69.\left(\frac{10.086+10.5(1-e^{-0.05z})}{16.7}\right)^{-n} \tag{S4}$$

4) AFM experiments were simulated using a finite element commercial software (ABAQUS). We employed an axisymmetric model in which the young's modulus of the gel was defined as a function of depth (given by Eq. S4), while the indentor was rigid (**Fig S7d**).

5) The force indentation curves obtained from AFM experiments were compared to that from the finite simulations for each guess (**Fig S7e**). The Young's modulus at the top of the gel and the corresponding exponent *n* would be adopted from the best fit (For the porous gel, E_{top}=135 Pa and *n*=1.0).

Quantifying the depth dependent Young's modulus before washing the EC monolayer

Having quantified the exponent *n*, from the decellularized gel, we next calculated the Young's modulus of the collagen matrix before decellularization and washing off the EC monolayer. To this end, the following steps were carried out:

1) Binarizing the confocal images taken from collagen fibers (before washing off the EC monolayer) and quantifying the mesh size at each focal plane (**Fig S8a, b**).

2) Calculation of the Young's modulus as a function of depth using the following equation (**Fig S8c**):

$$E = E_{bottom} \cdot \left(\frac{\zeta}{\zeta_{bottom}}\right)^{-n} \quad \text{(S5)}$$

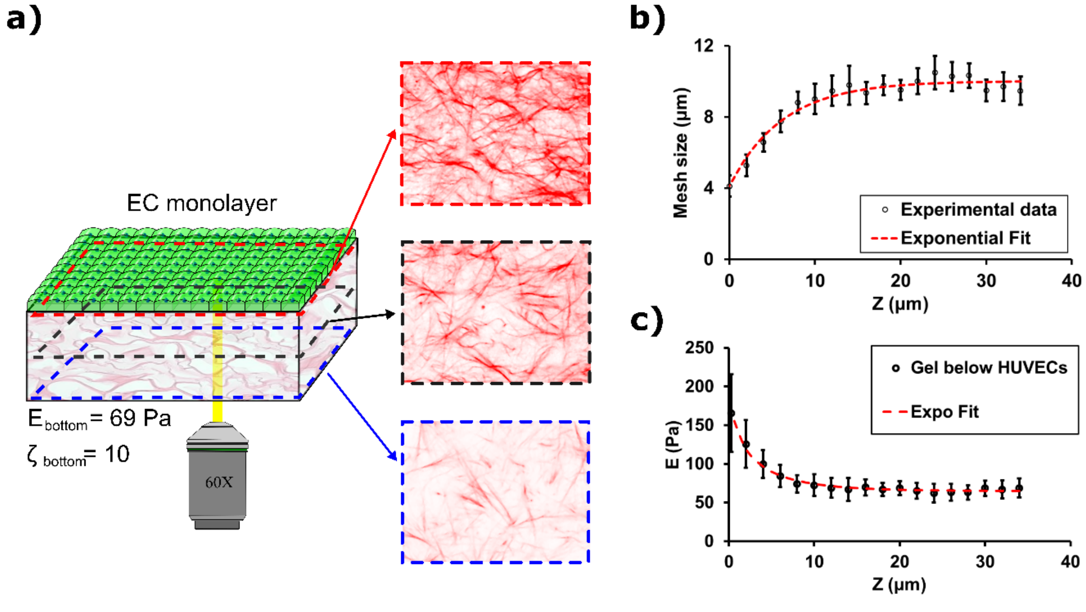

Fig S8. Quantification of the Young's modulus of the sub-endothelium matrix as a function of depth. **a)** Confocal images from the collagen fibers were analyzed at each elevation. **b)** Mesh size was calculated at each depth. **c)** Young's modulus was determined using the mesh size at each focal plane and the value of exponent *n* (For b and c; mean ±s.e.m, n= 8 samples).

Such procedures were repeated for control and HCC gels to characterize the mechanical properties of these gels as a function of depth.

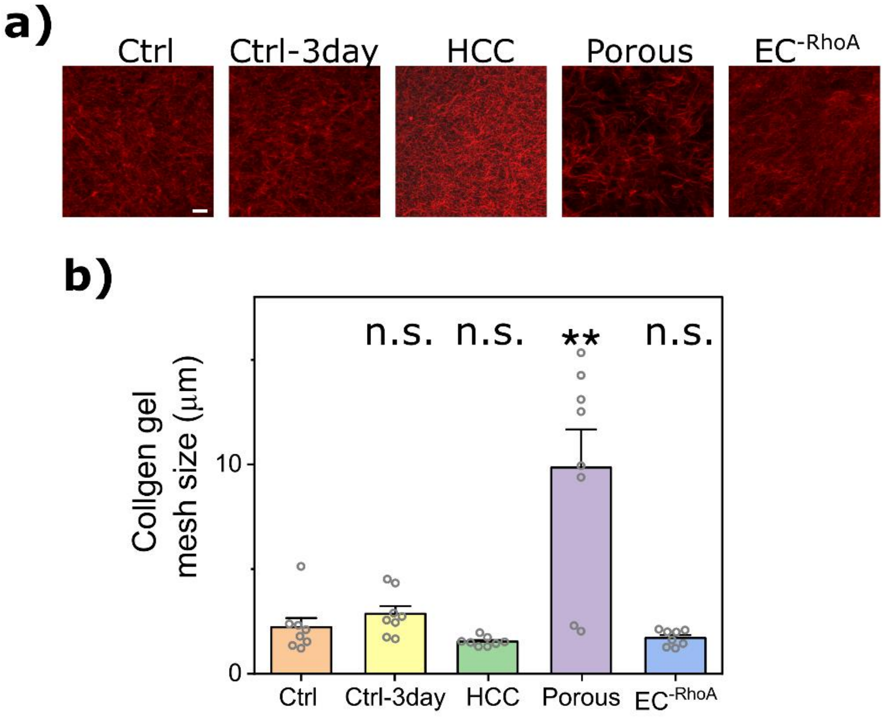

Fig S9. Quantification of mesh size of collagen gel for each condition. **a)** Collagen fibers below the endothelial monolayer for each condition. **b)** Comparison of mesh size for all conditions (n =8 samples, mean ± s.e.m.. Data points represent average mesh size of each sample obtained from 3 randomly selected ROIs, *p < 0.05 and **p<0.01, Student's t-test).

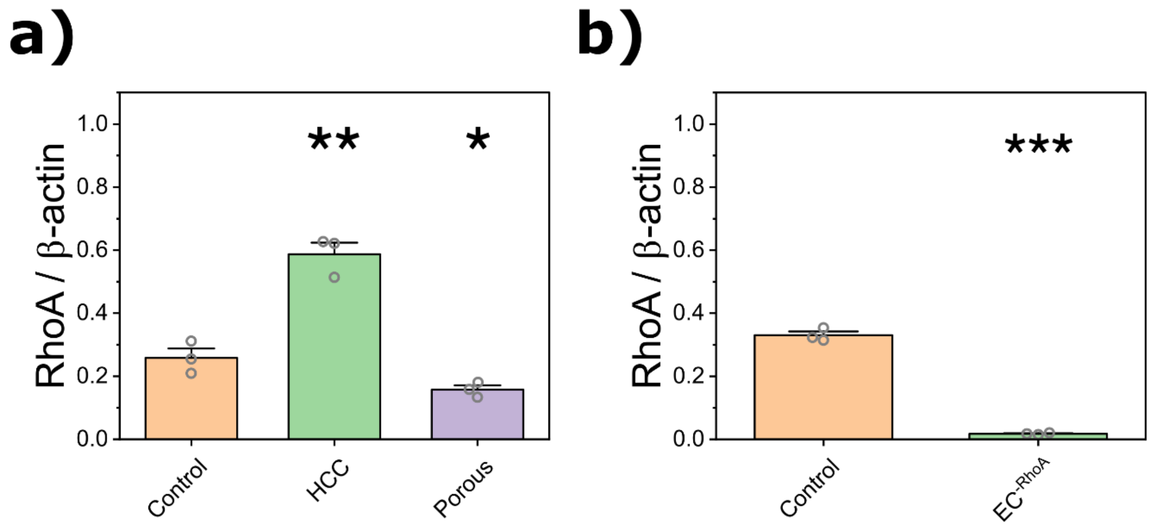

Fig S10) Relative levels of RhoA obtained from western blot analysis for **a)** Control, HCC, and Porous gels and **b)** Control and siRNA conditions (mean ± s.e.m., n =3. *p < 0.05, **p<0.01, ***p<0.001, Student's t-test).

Supplementary note 3- Staining myosin II

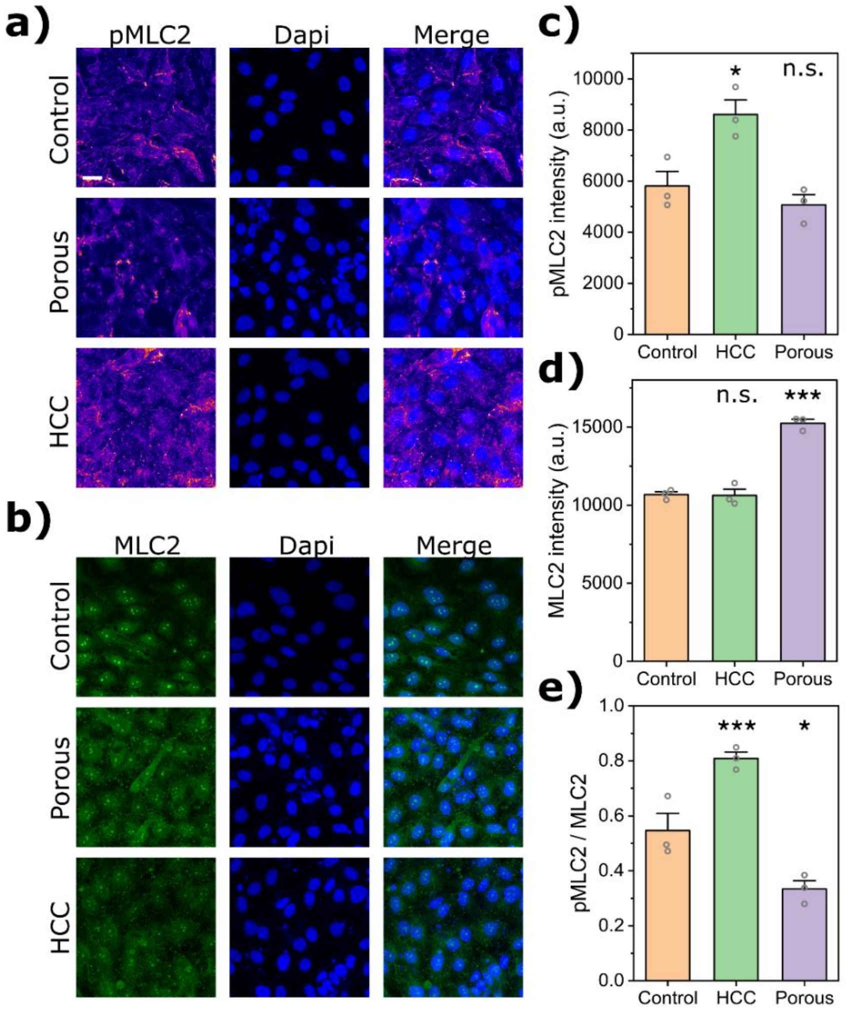

Fig S11) Impact of physical properties of subendothelial matrix on expression of **a)** phosphorylated myosin light chain 2 (pMLC2) and **b)** myosin light chain 2 (MLC2). Scale = 20 µm. **c)** Quantification of intensity of pMLC2 and **d)** MLC2 in different cases. **e)** Ratio of pMLC2 intensity to that for MLC2 in different cases. (For c, d, and e mean ± s.e.m., n=3 samples, in each sample (dish) 3 randomly selected regions were imaged using a confocal microscope; data points represent the average values calculated for each dish; *$p < 0.05$, **$p<0.01$, ***$p<0.001$, Student's t-test).

Immunofluorescence staining for MLC 2 and p-MLC2

Cells were fixed using 4% PFA at room temperature for 15 min. Cells were then washed in PBS, permeabilized for 10 min with 0.3% Triton-X in PBS and blocked for 1 h in blocking buffer (10% normal goat serum and 0.1% Triton-X in PBS). The primary antibodies were then applied overnight at 4 °C with gentle shaking. The following primary antibodies were used: Myosin Light Chain 2 (MLC2, Insight Biotechnology, 10906-1-AP) at a concentration 1:50 and Phospho-Myosin Light Chain 2 (pMLC2, Cell Signaling Technology, 3671S) at a concentration 1:50. Cells were then washed and incubated with secondary antibodies at room temperature for 2 h. The following secondary antibody was used: goat anti-rabbit (Cell Signaling Technology, 4412S) at a concentration of 1:500. Finally, cells were washed and stained with Hoechst 33342 for 10 min at room temperature. Samples were imaged using an upright confocal microscope (Zeiss Airyscan LSM 980).

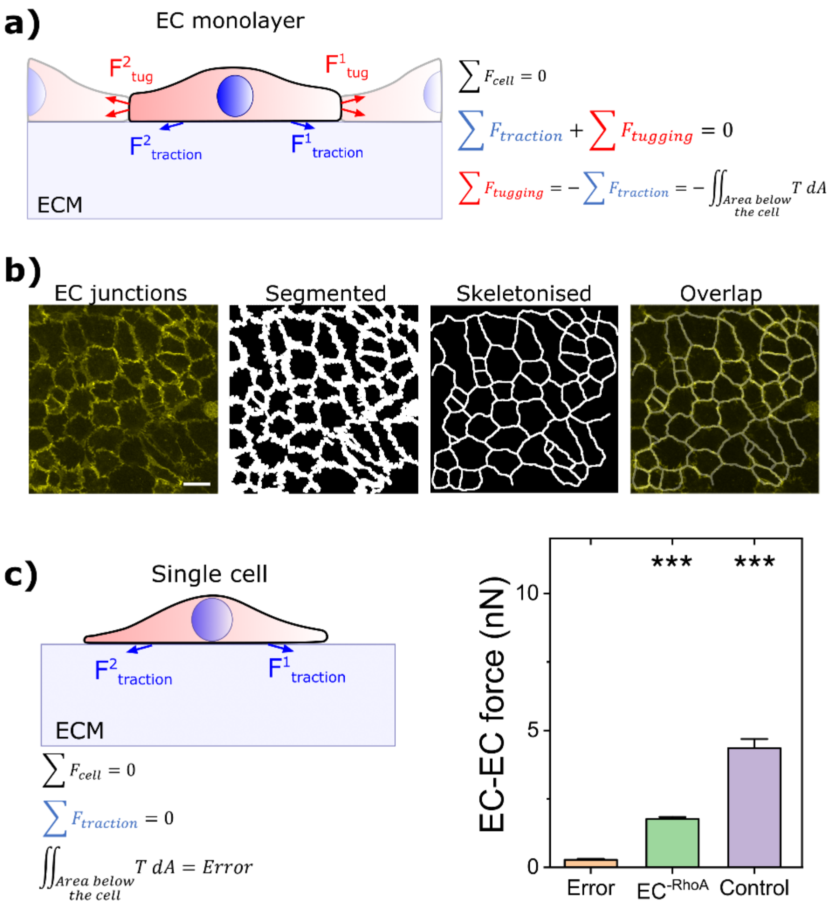

Fig S12. Quantification of EC-EC (tugging) forces. **a)** Since the dislocation of ECs in a monolayer is limited and gradual, it could be considered as a quasi-static process, in which cells are in force equilibrium at each time step and thus the net force acting on the cell should be zero. This implies that the sum of traction forces (EC-ECM interaction) and tugging forces (EC-EC interaction) should be zero and, therefore, the 'tugging forces' component is the negative integral of the traction forces (T) over the area covered by the cell. **b)** To find the area below the cell, we used the CD31 (yellow) channel to observe the boundaries of each cell. Then, after segmentation, the boundaries were skeletonized, and tractions were integrated inside each boundary. Cells in which their area was too big or too small were dismissed. **C)** Validation of the model. To quantify errors involving our force quantification technique, we quantified the forces generated by an isolated cell. Since the cell was stationary, the net force being applied to it should be zero. As there is no other cell in the vicinity of the cell, the tugging force is zero and the integral of traction forces should also be zero. Non-zero values show the level of error in our method which is 0.28 ±0.09 nN. The error is less than 16% of the minimum calculated forces (for EC-RhoA case) and less than 6.5% of forces measured for the control case (p-Value =1.3 e-6 and 4.7e-11, respectively; mean ± s.e.m, n ≥ 8 samples, ***p<0.001, Student's t-test)

Supplementary note 4- Details of the computer simulations

To simulate the mechanical interaction between the EC monolayer and ECM, we developed a computational model using the commercial finite element software ABAQUS in which a layer on ten cells was placed on top of a substrate. For the sake of simplicity, each cell was modelled as a rectangle while an ellipse was used to describe its nucleus. Typical values were chosen as the dimensions of cells (20 μm x 5 μm) and their nucleus (16 μm x 3 μm as the major and minor axes, respectively). Also, to avoid boundary effects, dimensions of the gel were selected to be sufficiently large (200 μm x 100 μm). Mesh size was also adopted after mesh sensitivity analysis. Additionally, the cells and the underlying gel were constrained in the lateral direction on the left, while the bottom of the gel was fixed in the vertical direction (**Fig S13a**). To simplify the model and reduce the computational time, a plane strain formulation was used to solve the boundary value problem. A linear elastic material model was employed to describe the mechanical properties of the gel and EC nuclei. A typical Young's modulus (500 Pa) and Poisson's ratio (0.4) were selected as the material properties of the EC nucleus, as these parameters were shown to have a negligible effect of the computational results. For the collagen substrate, Poisson's ratio was 0.2[24] while a range of values (100 – 500 Pa), that covers the stiffness of the collagen gels used in this study, was selected as its Young's modulus. To describe the mechanical properties of the cytoskeleton, a model developed by Shenoy et al.[25] was used, in which cells are considered as materials with passive and active properties. As a passive material, cells would deform under external forces while the active element enables them to apply contractile forces to their surrounding microenvironment. To consider the passive behaviour, the model uses two parameters (bulk modulus, K, and shear modulus, μ) while to accommodate the active element, the model requires three more parameters (motor density in the quiescent state, ρ_0, chemical stiffness, β, and chemo-mechanical feedback parameter, α). ρ_0 shows the contractile ability of an isolated cell in the absence of ECM or any other cell, β controls the effect of molecular mechanisms that regulate the engagement of motors, while α is responsible for the feedbacks affecting the stress-dependent signalling pathways. As porosity of the ECM regulates actin polymerization and RhoA signalling, its effect was taken into account through changing the feedback parameterα. RhoA activity and cell generated force are less, when we use a porous substrate, thus a lower a should be used for more porous matrix. All the cytoskeletal material parameters were taken from Shenoy et al.[25], except β, as a slight increase in its value led to us obtaining more accurate results. Material parameters are also shown in (**Fig S13a**).

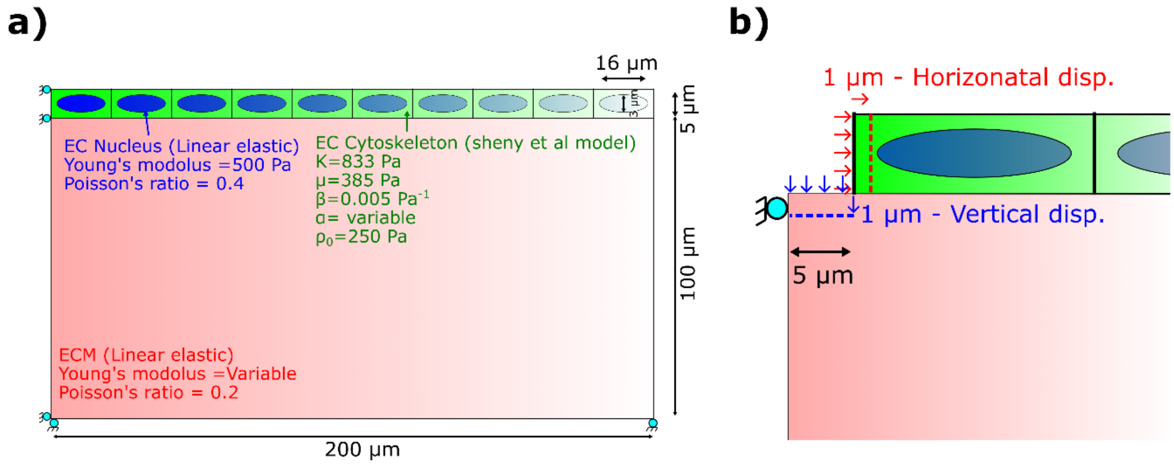

Fig S13. Computer simulation details. **a)** Geometry, boundary condition, material models, and material parameters used for computer simulation in ABAQUS. **b)** Quantifying horizontal and vertical stiffness (i.e., force required to generated unit displacement)

Formulation of the model for 3D condition has been provided previously by Shenoy et al.[25], briefly:

$$\sigma_{ij} = \frac{1}{3}\sigma_{kk}\delta_{ij} + \tilde{\sigma}_{ij} \qquad \rho_{ij} = \frac{1}{3}\rho_{kk}\delta_{ij} + \tilde{\rho}_{ij}$$
$$\sigma_{kk} = 3\rho_0 + 3\bar{K}\varepsilon_{kk} \qquad \rho_{kk} = 3\bar{\rho}_0 + 3\bar{K}_\rho\varepsilon_{kk} \tag{s6}$$
$$\tilde{\sigma}_{ij} = 2\bar{\mu}\tilde{\varepsilon}_{ij} \qquad \tilde{\sigma}_{ij} = 2\bar{\mu}_\rho\tilde{\varepsilon}_{ij}$$

in which, σ_{ij} is the stress tensor, δ_{ij} is the Kronecker delta and ρ_{ij} represents the contractility tensor. $\sigma_{kk} = \sigma_{11} + \sigma_{22} + \sigma_{33}$ and $\varepsilon_{kk} = \varepsilon_{11} + \varepsilon_{22} + \varepsilon_{33}$ where ε_{ij} is the strain tensor.

$$\bar{\rho}_0 = \frac{\beta\rho_0}{\beta - \alpha} \qquad \bar{K} = \frac{K\beta - {}^1\!/_3}{\beta - \alpha} \tag{S7}$$
$$\bar{\mu} = \frac{\mu\beta - {}^1\!/_2}{\beta - \alpha} \qquad \bar{K}_\rho = \frac{K\alpha - {}^1\!/_3}{\beta - \alpha} \qquad \bar{\mu}_\rho = \frac{\mu\alpha - {}^1\!/_2}{\beta - \alpha}$$

For the plane strain case, the formulation can be simplified as below:

$$\begin{Bmatrix}\sigma_{11}\\ \sigma_{22}\\ \sigma_{33}\\ \sigma_{12}\end{Bmatrix} = \bar{\rho}_0\begin{Bmatrix}1\\1\\1\\0\end{Bmatrix} + \begin{bmatrix}\bar{K} + \frac{4}{3}\bar{\mu} & \bar{K} - \frac{2}{3}\bar{\mu} & 0\\ \bar{K} - \frac{2}{3}\bar{\mu} & \bar{K} + \frac{4}{3}\bar{\mu} & 0\\ \bar{K} - \frac{2}{3}\bar{\mu} & \bar{K} - \frac{2}{3}\bar{\mu} & 0\\ 0 & 0 & \bar{\mu}\end{bmatrix}\begin{Bmatrix}\varepsilon_{11}\\ \varepsilon_{22}\\ \gamma_{12}\end{Bmatrix} \qquad \begin{Bmatrix}\rho_{11}\\ \rho_{22}\\ \rho_{33}\\ \rho_{12}\end{Bmatrix} = \bar{\rho}_0\begin{Bmatrix}1\\1\\1\\0\end{Bmatrix} + \begin{bmatrix}\bar{K}_\rho + \frac{4}{3}\bar{\mu}_\rho & \bar{K}_\rho - \frac{2}{3}\bar{\mu}_\rho & 0\\ \bar{K}_\rho - \frac{2}{3}\bar{\mu}_\rho & \bar{K}_\rho + \frac{4}{3}\bar{\mu}_\rho & 0\\ \bar{K}_\rho - \frac{2}{3}\bar{\mu}_\rho & \bar{K}_\rho - \frac{2}{3}\bar{\mu}_\rho & 0\\ 0 & 0 & \bar{\mu}_\rho\end{bmatrix}\begin{Bmatrix}\varepsilon_{11}\\ \varepsilon_{22}\\ \gamma_{12}\end{Bmatrix} \tag{S8}$$

where $\gamma_{12} = 2\varepsilon_{12}$. The constitutive model (i.e., SI Eq. 2 and 3) were implemented into ABAQUS using a UMAT developed in Fortran.

To obtain the horizontal and vertical stiffnesses, i.e., the forces required to generate unit displacements either in lateral or vertical directions, we modified the geometry slightly, while the other dimensions, material models and properties remained unchanged. We created a 5 µm gap on the left-hand side of the monolayer; this allowed us to apply a 1 µm horizontal displacement to the left of the monolayer and measure the concomitant reaction forces. Furthermore, we applied a 1 µm vertical displacement to the gap and quantified the vertical stiffness of the matrix (**Fig S13b**).

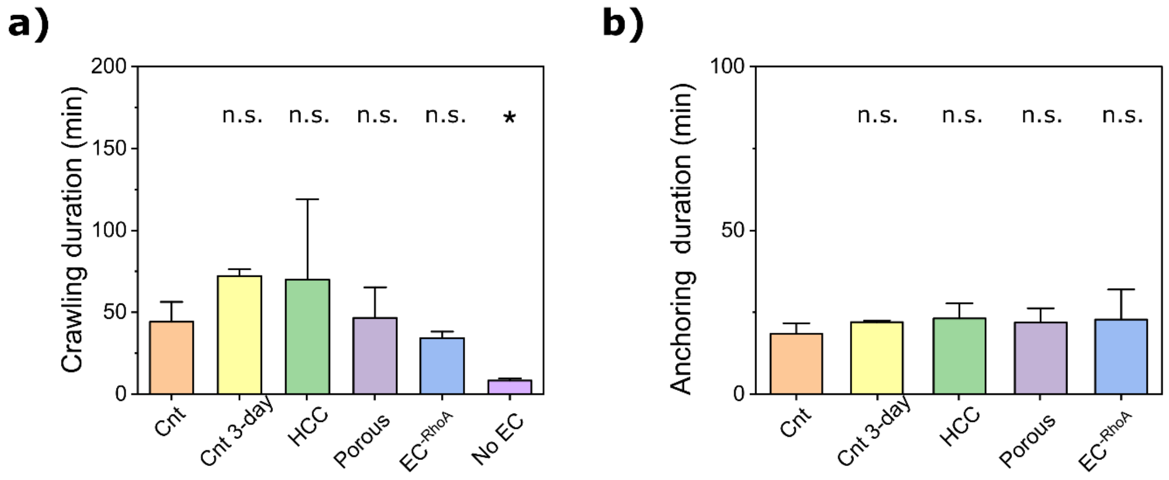

Fig S14. Duration of **a)** crawling stage and **b)** initial protrusion stage for different cases. For "No EC" case the crawling stage starts by introduction of TCs to the top of matrix and terminates when TCs stop moving on the matrix and firmly attach to it. (data are mean ± s.e.m., n ≥ 3 samples, **p* < 0.05, Student's t-test).

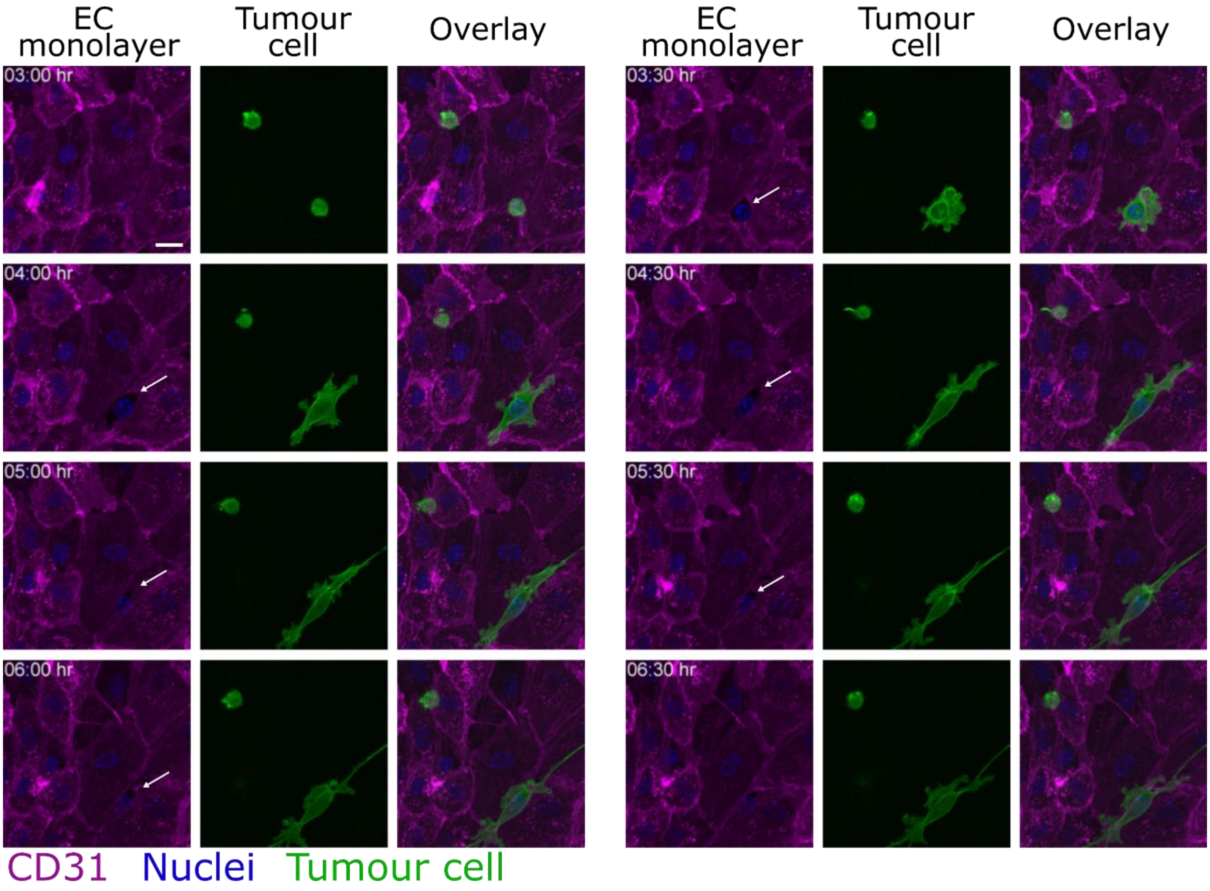

Fig S15) Time-lapse confocal images representing the disruption of endothelial junctions and formation of a gap during TEM and its closure after TC transmigration. White arrows indicate the location of the gap. The nuclei located in the gap belongs to the TC. Scale = 20 µm.

Supplementary Note 5- Mechanics of cancer extravasation

The aim of this note is to illustrate the traction distribution on a representative cell for each condition. Also, average tractions and the duration were quantified for each stage.

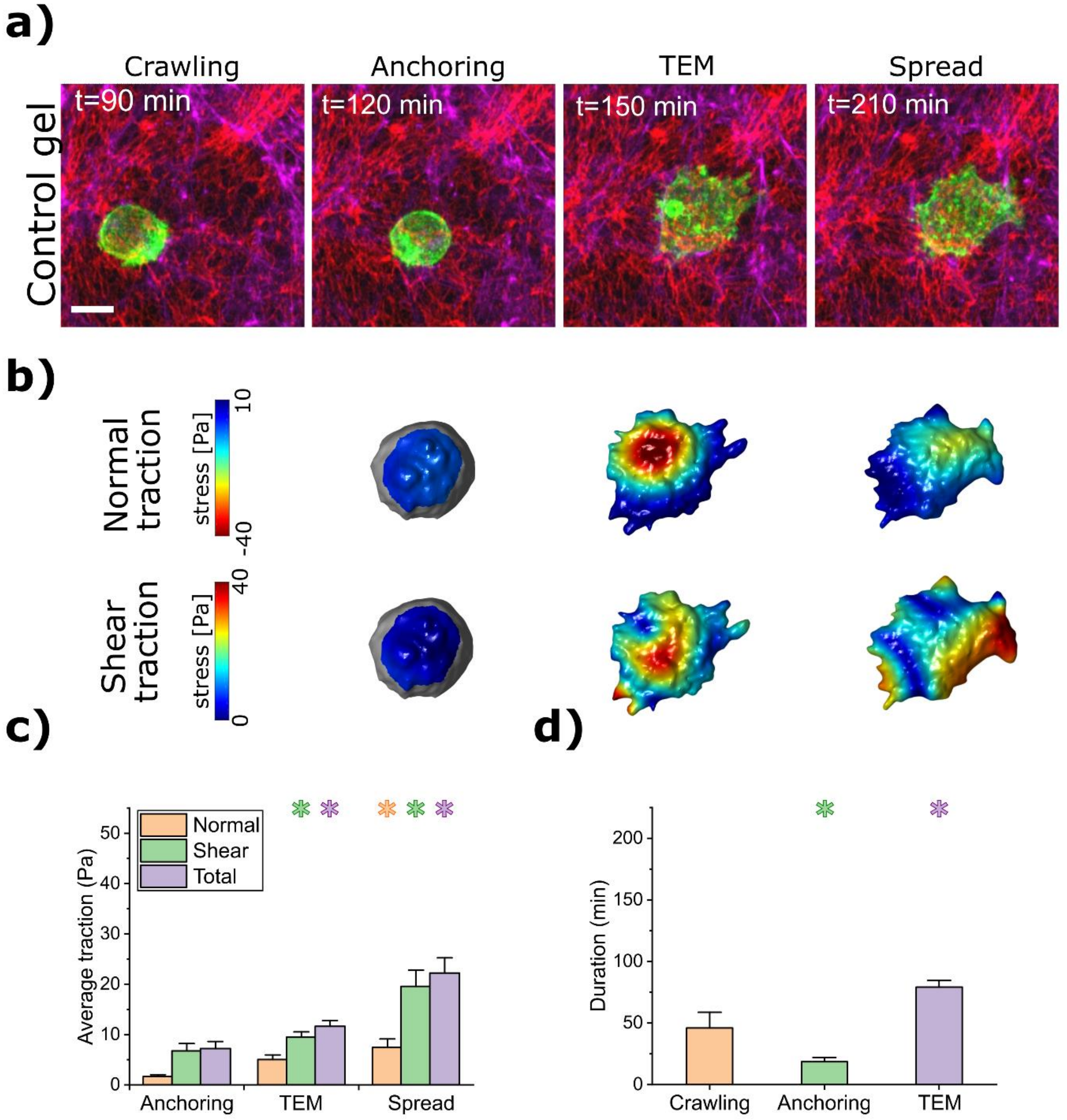

Fig S16. Mechanics of extravasation for the "Control" case. **a)** Confocal Images showing different stages of extravasation (TC, green; EC monolayer, LifeAct in magenta; Collagen matrix, red). Scale = 20 µm **b)** Incremental normal and shear tractions acting on the interface of the TC and subendothelial matrix. For the normal traction, negative sign means the TC pushes down on the matrix. **c)** Average of cumulative normal, shear, and total tractions acting on the TC interface during different stages of extravasation. **d)** Duration of each stage. (For c and d, data are mean ± s.e.m., n≥10 dishes, **p* < 0.05, ***p*<0.01, and ****p*<0.001, Student's t-test. Data compared with the "Anchoring" and "Crawling" stages in c and d, respectively).

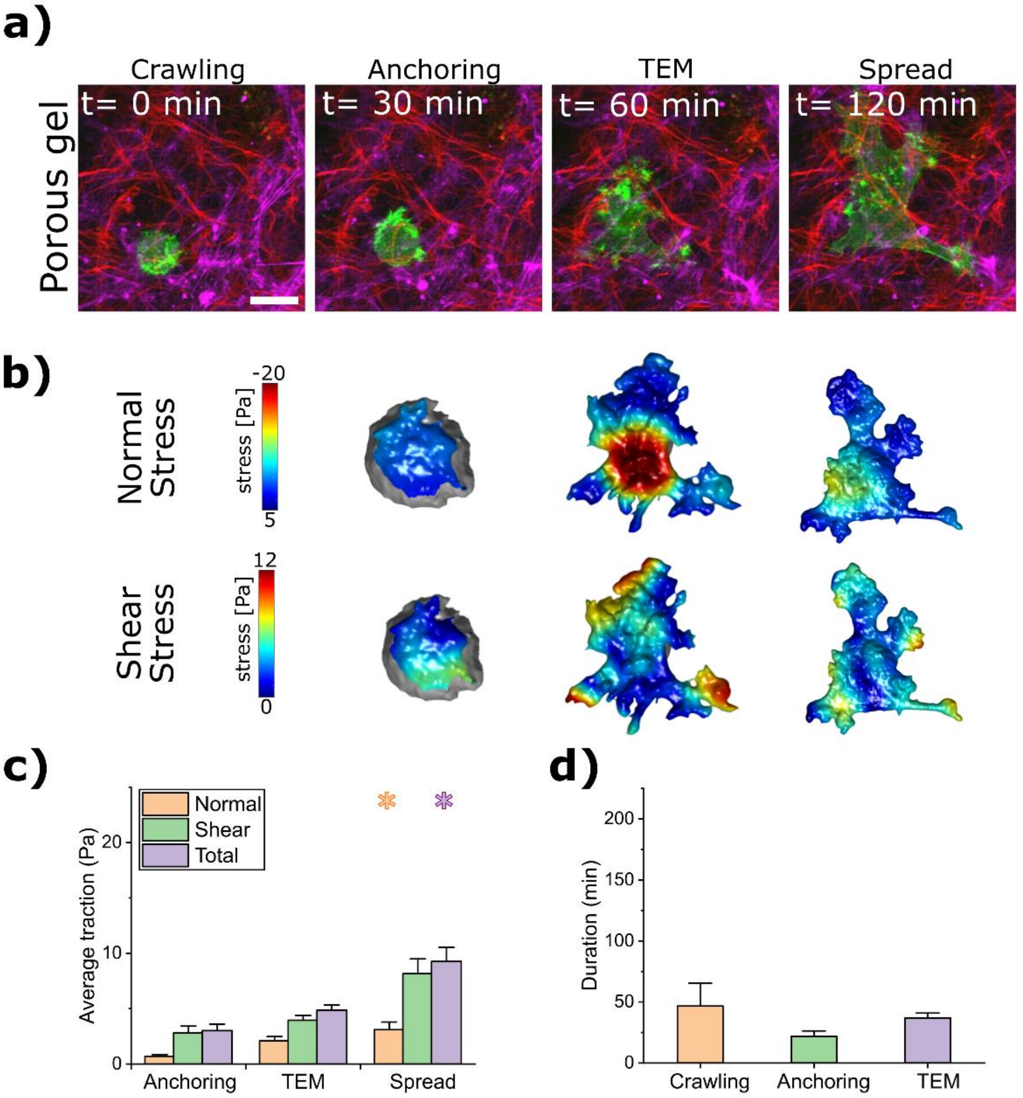

Fig S17. Mechanics of extravasation for the "Porous" case. **a)** Confocal images showing different stages of extravasation (TC, green; EC monolayer, LifeAct in magenta; Collagen matrix, red). Scale = 20 µm **b)** Incremental normal and shear tractions acting on the interface of the TC and subendothelial matrix. For the normal traction, negative sign means the TC pushes down on the matrix. **c)** Average of cumulative normal, shear, and total tractions acting on the TC interface during different stages of extravasation. **d)** Duration of each stage (For c and d, data are mean ± s.e.m., n ≥ 4 samples, *$p < 0.05$, Student's t-test. Data compared with the "Anchoring" and "Crawling" stage in c and d, respectively).

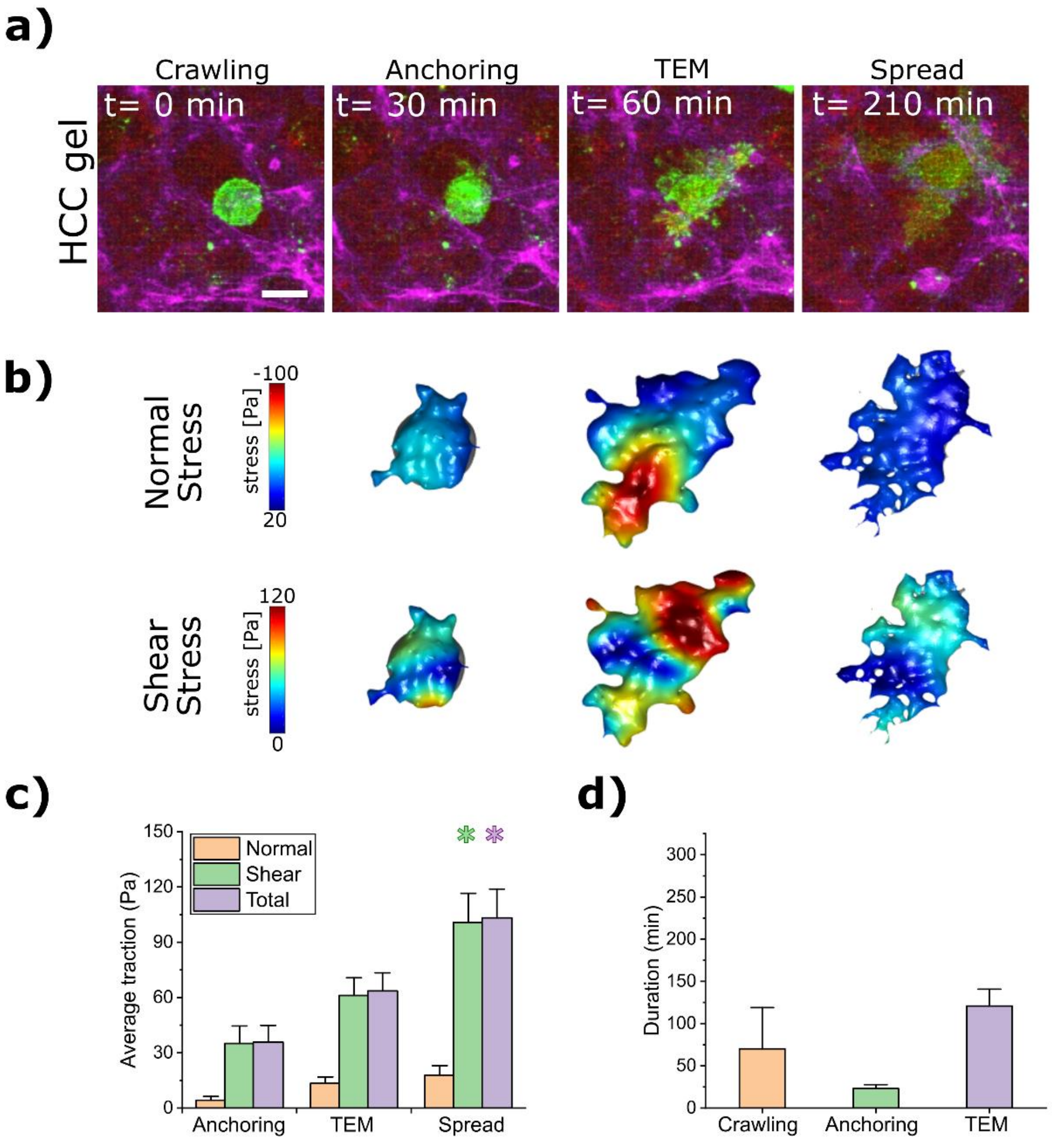

Fig S18. Mechanics of extravasation for the "high collagen content (HCC)" case. **a)** Confocal images showing different stages of extravasation (TC, green; EC monolayer, LifeAct in magenta; Collagen matrix, red). Scale = 20 µm **b)** Incremental normal and shear tractions acting on the interface of the TC and subendothelial matrix. For the normal traction, negative sign means the TC pushes down on the matrix. **c)** Average of cumulative normal, shear, and total tractions acting on the TC interface during different stages of extravasation. **d)** Duration of each stage (For c and d, data are mean ± s.e.m., n ≥ 4 samples, **p* < 0.05, Student's t-test. Data compared with the "Anchoring" and "Crawling" stage in c and d, respectively).

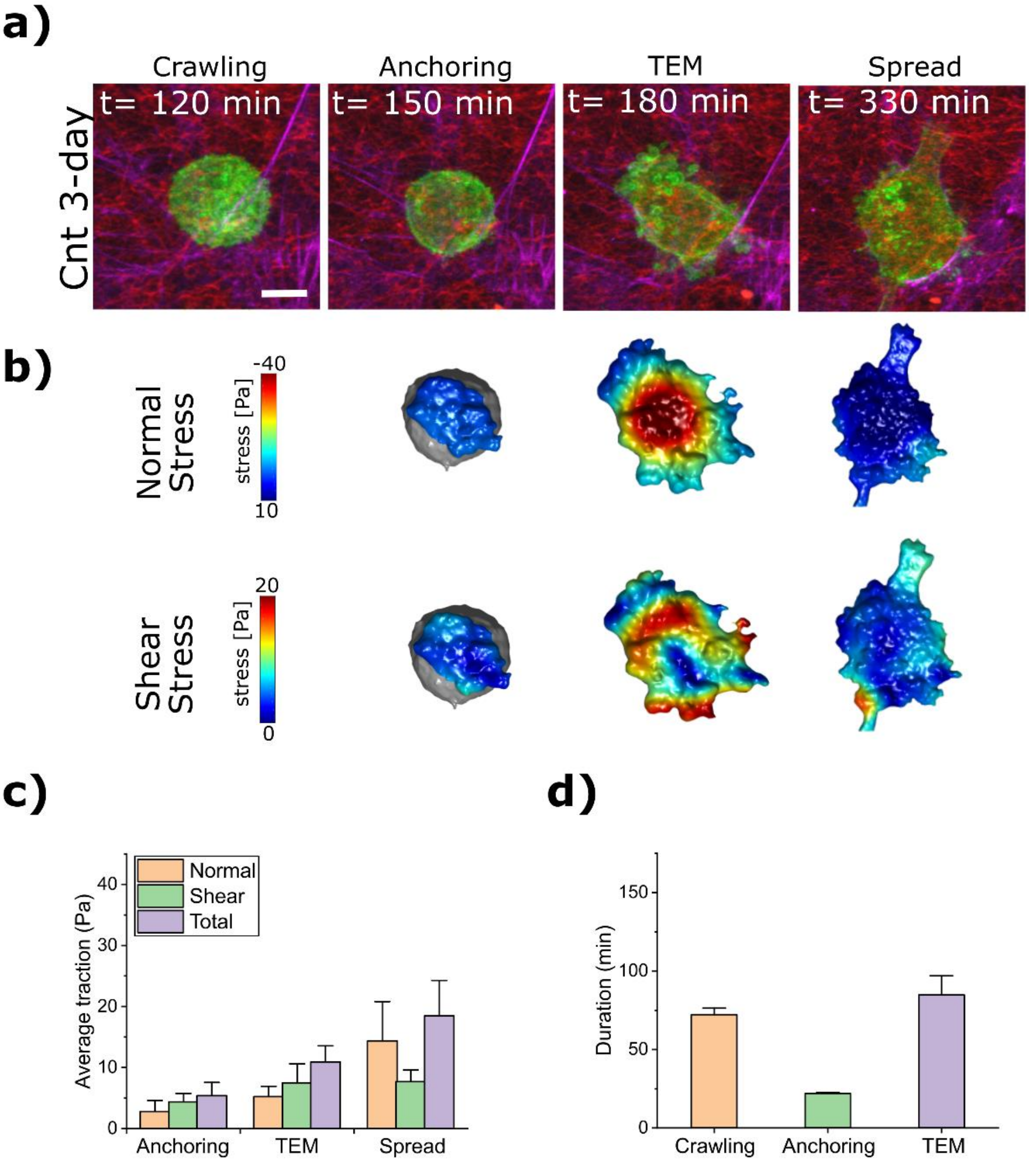

Fig S19. Mechanics of extravasation for the "Cnt 3-day" case. **a)** Confocal images showing different stages of extravasation (TC, green; EC monolayer, LifeAct in magenta; Collagen matrix, red). Scale = 20 µm **b)** Incremental normal and shear tractions acting on the interface of the TC and subendothelial matrix. For the normal traction, negative sign means the TC pushes down on the matrix, **c)** Average of cumulative normal, shear, and total tractions acting on the TC interface during different stages of extravasation. **d)** Duration of each stage (For c and d, data are mean ± s.e.m., n ≥ 4 samples, *$p < 0.05$, Student's t-test. Data compared with the "Anchoring" and "Crawling" stage in c and d, respectively).

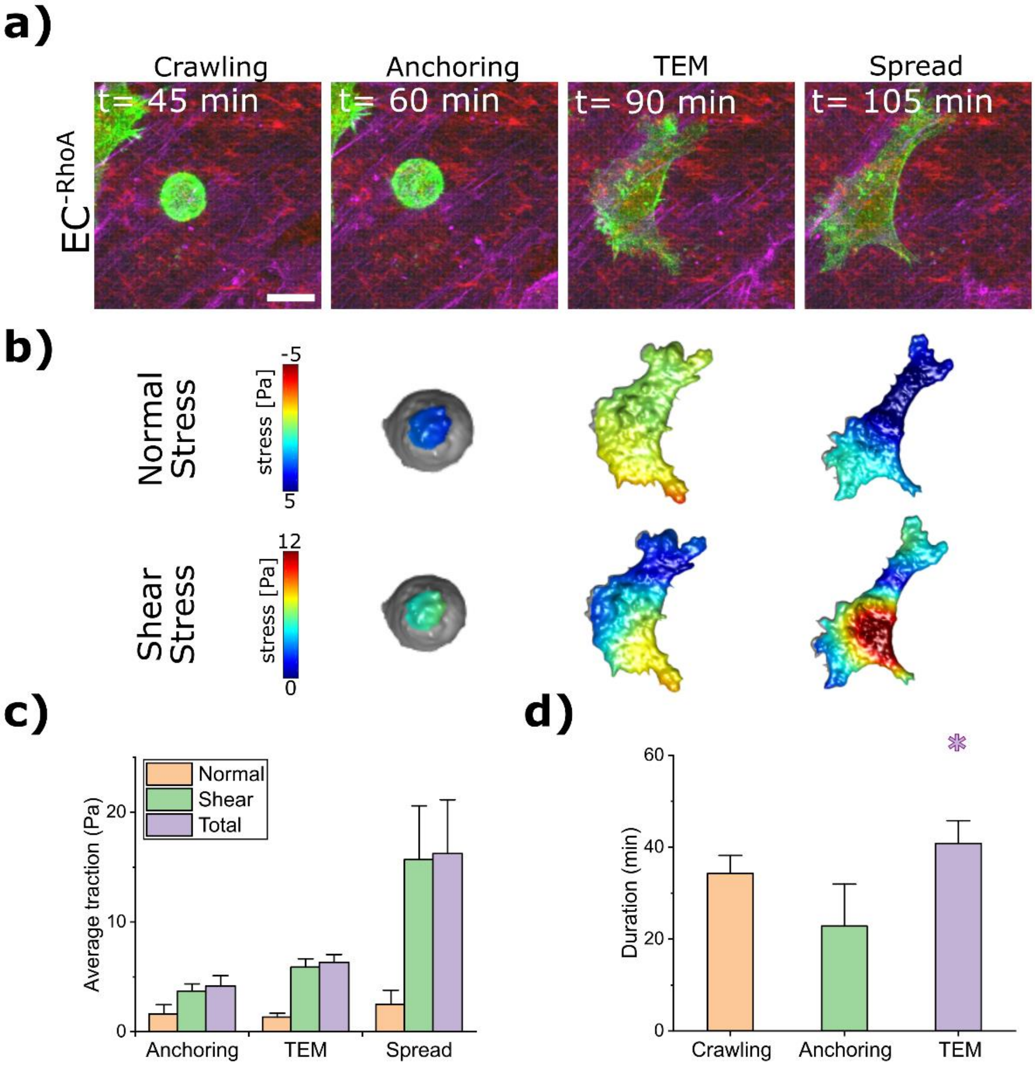

Fig S20. Mechanics of extravasation for the "EC^{-RhoA}" case. **a)** Confocal images showing different stages of extravasation (TC, green; EC monolayer, LifeAct in magenta; Collagen matrix, red). Scale = 20 µm **b)** Incremental normal and shear tractions acting on the interface of the TC and subendothelial matrix. For the normal traction, negative sign means the TC pushes down on the matrix. **c)** Average of cumulative normal, shear, and total tractions acting on the TC interface during different stages of extravasation. **d)** Duration of each stage (For c and d, data are mean ± s.e.m., n ≥ 3 samples, **p* < 0.05, Student's t-test. Data compared with the "Anchoring" and "Crawling" stage in c and d, respectively).

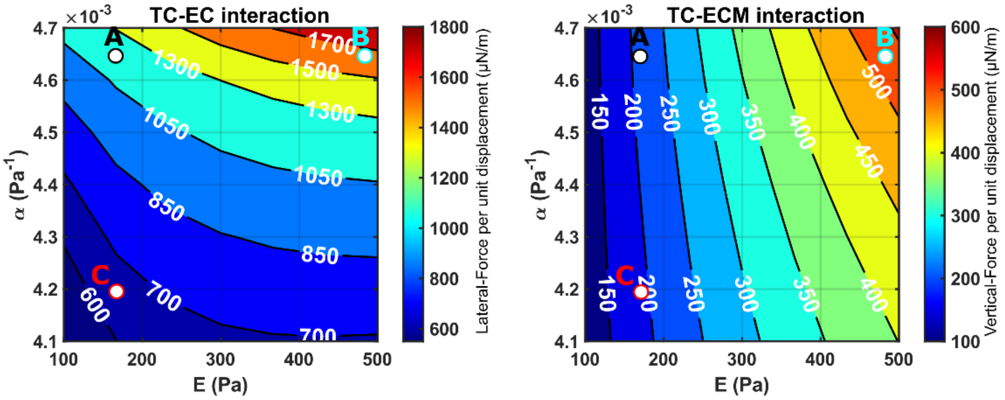

Fig S21. Impact of stiffness of the collagen substrate (E) and its porosity (through feedback parameter a) on the lateral stiffness of EC monolayer (i.e., the force that needs be applied to ECs to generate unit lateral displacement in the monolayer) and vertical stiffness (i.e., the force that needs be applied to generate unit vertical displacement in ECM) obtained from computer simulations. Points A, B, and C denote conditions corresponding to control, high collagen content (HCC), and porous cases, respectively. The same feedback parameter a (4.64E-3 Pa^{-1}) was used to simulate the behaviour of collagen gel in control (point A) and HCC (point B) conditions as the mesh size is not significantly different for these cases. However, since for the porous condition, the mesh size is larger than that for the control, a smaller a (4.2E-3 Pa^{-1}) was employed for that case. Additionally, the Young's modulus of collagen gel for the control and porous gel was in same range (164 Pa), while a higher value (460 Pa) was used for the HCC condition. The contour plots show that the lateral stiffness of the monolayer for the HCC case (~1700 µN/m) is larger than that for the control case (~1100 µN.m) while the monolayer formed on the porous gel has the minimum lateral stiffness (~650 µN/m). Similarly, the vertical stiffness of the gel in the HCC case (~525 µN/m) is more than that for the control case (~210 µN/m) and the minimum vertical stiffness belong to the porous case (~190 µN/m).

Supplementary Note 6- Impact of EC density

For all experiments in this study, EC monolayers were formed by introducing 350,000 HUVECs to the MatTek dish. We chose this EC density after optimizing the culturing protocol for ECs at different densities (2.e5, 3.e5, 3.5e5, and 4.e5) and investigating the confluency and integrity of the monolayer after 48 hrs. Using standard T75 flasks, we estimated that the initial cell density should be ~3. – 4.e5 per dish, since ~3e6 cells provide a confluent monolayer in the flask, and the area of our dish is ~10.2 cm^2 (~7 times less than a T75 flask). Considering the proliferation rate of ECs, using an initial density of ~3. – 4.e5 cells should result in a seamless confluent monolayer after 48 hrs. The results showed that with 3.e5 cells, there were some non-confluent regions in the dish, while for the density of 5.e5, we found some overconfluent regions (**Fig S22**).”

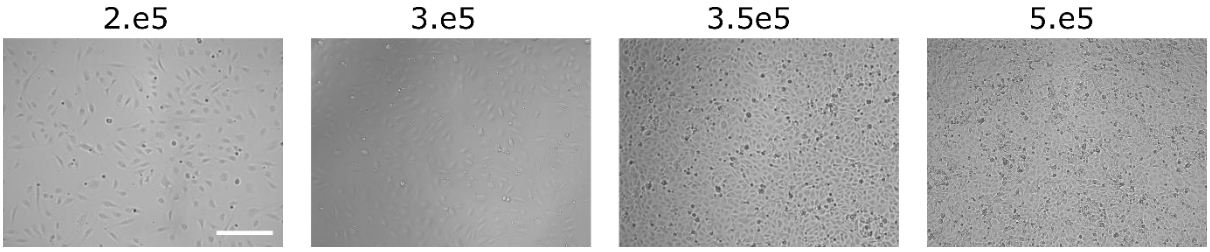

Fig S22) Effect of endothelial cell density on confluency of the monolayer. The monolayer with 2e5 and 3e5 initial cell densities, was not fully confluent, while for 5e5 cells, some regions were overconfluent. Using a cell density of 3.5e5, a seamless confluent EC monolayer was formed after 48 hr of culture. Scale = 200 µm.

To investigate the impact of cell density on transmigration potential, we formed EC monolayers with two different initial densities: 3.5e5 and 7e5. The results revealed that for the higher EC density, transmigration rate and efficiency decreased significantly, implying that it is more difficult for TCs to transmigrate through a compacter monolayer (**Fig S23**).

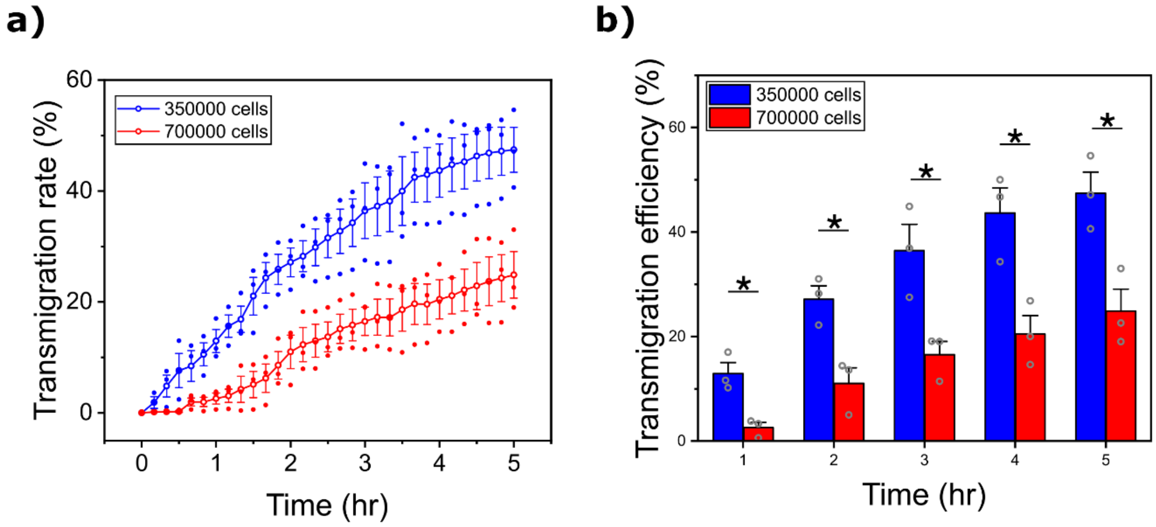

Fig S23) Effect of EC density on **a)** transmigration rate and **b)** efficiency (mean ± s.e.m., n=3 samples (dishes); data points represent the average transmigration rate / efficiency for each dish. *$p < 0.05$, Student’s t-test).

Supplementary note 7 - Verification of the force microscopy method

The aim of this note is to evaluate the accuracy of the method used to quantify the cell-generated tractions. The protocol included the following steps:

1- Calculating the Young's modulus of the subendothelial matrix as a function of depth, (**supplementary note 2**).

2- Taking time-lapse confocal images of the collagen fibers of the subendothelial matrix. At least two time-points, *t* and $t+\Delta t$, are required.

3- Measuring the displacement field in the matrix between *t* and $t+\Delta t$ using the code developed by Bar-Kochaba et al.[26].

4- Calculating the increment of cell-generated tractions between *t* and $t+\Delta t$, by implementing the data obtained from the two previous steps into a commercial finite element software (ABAQUS).

Here, we used a collagen gel (6 mg/ml, incubated at 37°C for 30 min) and quantified its Young's modulus as a function of depth after washing the EC monolayer, using the method explained in Supplementary note 2. The Young's modulus and mesh size were 300 Pa and 1.67 at the bottom of the gel, respectively. Considering the mesh size at the top (ξ=1.34), the Young's modulus was calculated at the top (E_{top} = 700 Pa, **Fig S24**).

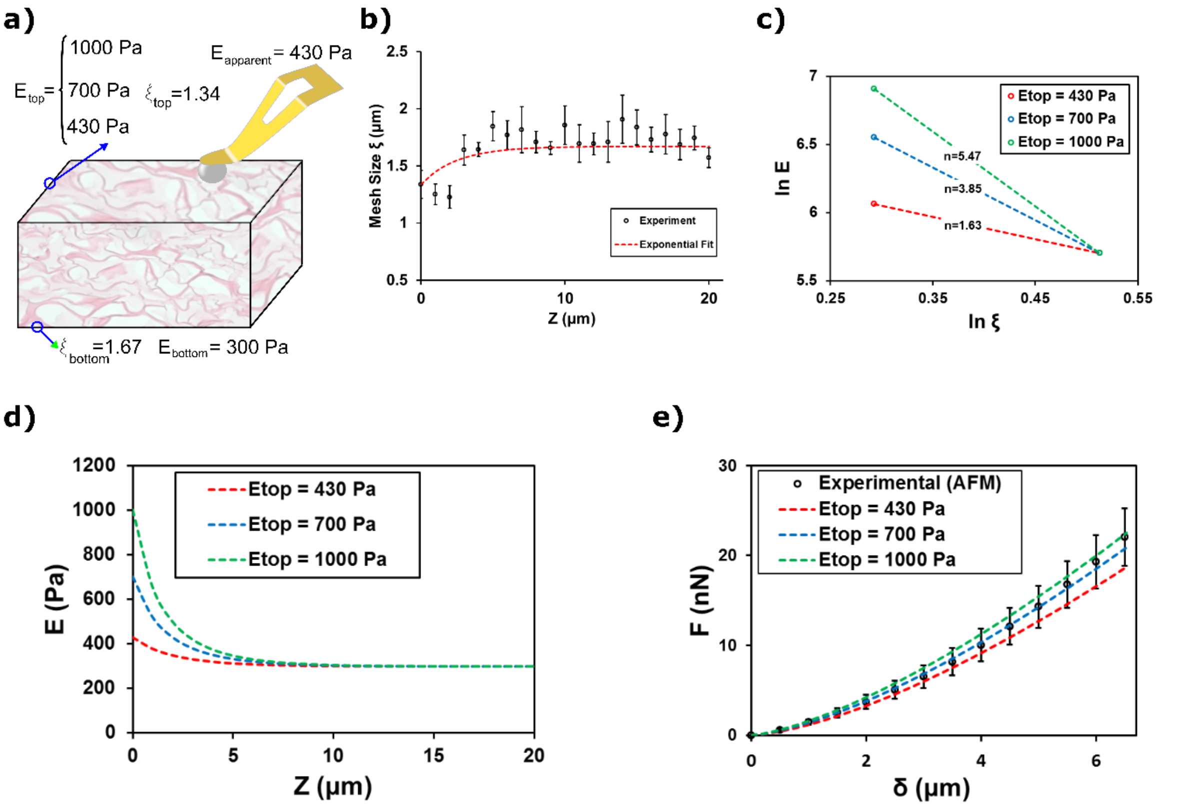

Fig S241. Determination of the Young's modulus at the top and the material parameter (exponent *n*). **a)** The Young's modulus at the top of the gel was quantified by trial and error. Three typical guesstimates are shown for E_{top}. b) Calculating the mesh size as a function of depth (data are mean ± s.e.m, n=8 ROIs from three independent samples). **c)** Determination of the exponent *n* for each guess. **d)** Finding the variation in the Young's modulus as a function of depth for each guess. **e)** Comparison of the force-indentation curve obtained from FEA simulations and AFM experiments (mean ±s.e.m, n= 4 indentation tests).

To verify the accuracy of the method, we must apply a known force to the matrix, then follow the above-mentioned steps to estimate the force and compare the result with the applied force. To this end, we employed AFM experiments to indent the gel shown in **Fig S25**, using a spherical tip glued to the end of cantilever. The sample stage of the AFM was mounted on an inverted confocal microscope, allowing us to image fluorescently labelled collagen fibers at the same time. We applied a 35 nN force to the gel which caused 8.2 μm indentation depth. Then, the following steps were carried out to reconstruct the force:

1) Confocal images of the collagen fibers were captured (**Fig S25a**), 2) Calculating the displacements using the FIDVC code (**Fig S25b**), 3) Performing the finite element simulation and estimating the stress distribution on the AFM tip (**Fig S25c**) and, 4) Integrating the stresses to measure the total force applied by the AFM tip to the matrix and compare the reconstructed force with AFM measurements (**Fig S25d**).

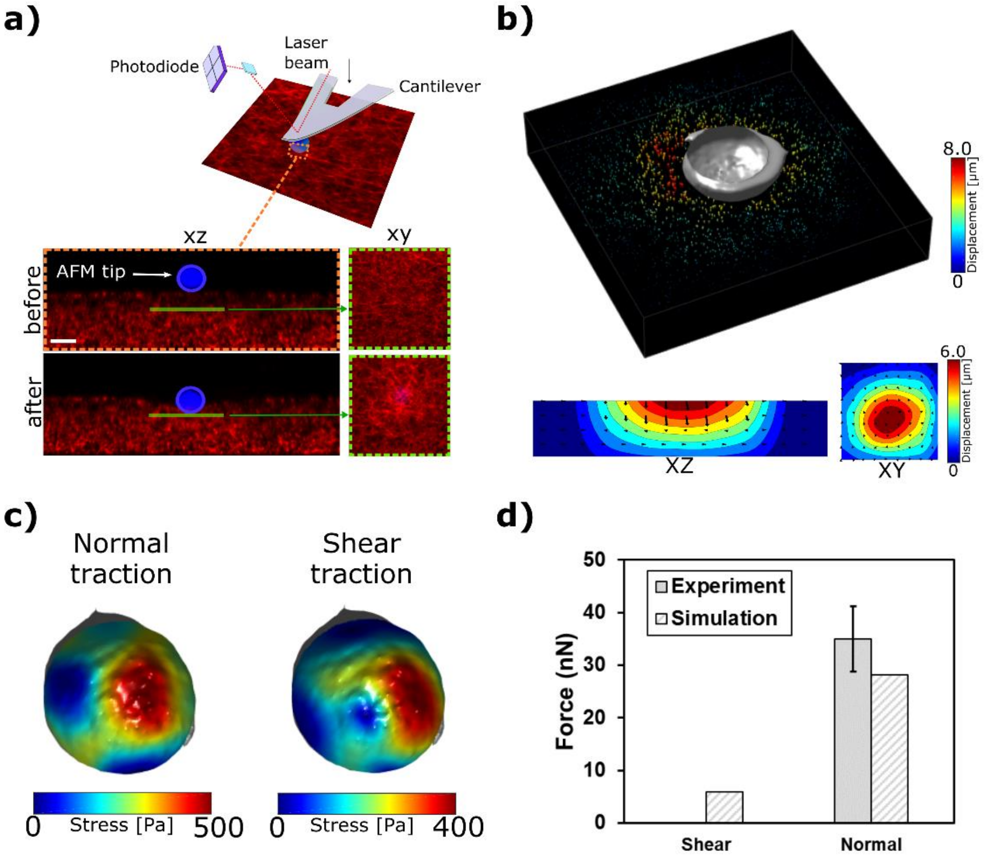

Fig S25. Quantifying the forces applied to the AFM tip. **a)** Confocal images from the hydrogel before and after AFM indentation. **b)** Displacement pattern in the gel. **c)** Stress distribution on the AFM tip. **d)** Comparison of normal and shear forces applied in the AFM experiment with the reconstructed forces (data are mean ± S.D, n=4 experiments).

Comparison of the reconstructed normal (28.1 nN) and shear (5.9 nN) forces with the applied forces (35 nN and 0 nN, respectively) shows that the error in prediction of forces could be up to 20%, which is within an acceptable range.

References:

[1] M. L. Chen, J. W. Ruberti, T. D. Nguyen, *J. Mech. Behav. Biomed. Mater.* **2018**, *82*, 345.
[2] B. T. Wilks, E. B. Evans, A. Howes, C. M. Hopkins, M. N. Nakhla, G. Williams, J. R. Morgan, *Adv. Sci.* **2022**, *9*, 2103939.
[3] S. O. Sarrigiannidis, J. M. Rey, O. Dobre, C. González-García, M. J. Dalby, M. Salmeron-Sanchez, *Mater. Today Bio* **2021**, *10*, 100098.
[4] B. N. Mason, A. Starchenko, R. M. Williams, L. J. Bonassar, C. A. Reinhart-King, *Acta Biomater.* **2013**, *9*, 4635.
[5] H. M. Powell, S. T. Boyce, *Biomaterials* **2006**, *27*, 5821.
[6] G. M. Fernandes-Cunha, K. M. Chen, F. Chen, P. Le, J. H. Han, L. A. Mahajan, H. J. Lee, K. S. Na, D. Myung, *Sci. Rep.* **2020**, *10*, 16671.
[7] A. Rajaram, C. C. Chu, *J. Biomater. Sci. Polym. Ed.* **1989**, *1*, 167.
[8] M. J. A. van Luyn, P. B. van Wachem, L. Olde Damink, P. J. Dijkstra, J. Feijen, P. Nieuwenhuis, *J. Biomed. Mater. Res.* **1992**, *26*, 1091.
[9] J. G. Snedeker, A. Gautieri, *Muscles. Ligaments Tendons J.* **2014**, *4*, 303.
[10] M. Nair, R. K. Johal, S. W. Hamaia, S. M. Best, R. E. Cameron, *Biomaterials* **2020**, *254*, 120109.
[11] G. S. Shannon, T. Novak, C. Mousoulis, S. L. Voytik-Harbin, C. P. Neu, *RSC Adv.* **2015**, *5*, 2113.
[12] G. C. Wood, M. K. Keech, *Biochem. J.* **1960**, *75*, 588.
[13] Y.-J. Hwang, J. G. Lyubovitsky, *Anal. Methods* **2011**, *3*, 529.
[14] K. A. Jansen, A. J. Licup, A. Sharma, R. Rens, F. C. MacKintosh, G. H. Koenderink, *Biophys. J.* **2018**, *114*, 2665.
[15] C. B. Raub, V. Suresh, T. Krasieva, J. Lyubovitsky, J. D. Mih, A. J. Putnam, B. J. Tromberg, S. C. George, *Biophys. J.* **2007**, *92*, 2212.
[16] J. Xie, M. Bao, S. M. C. Bruekers, W. T. S. Huck, *ACS Appl. Mater. Interfaces* **2017**, *9*, 19630.
[17] P. V. Taufalele, J. A. VanderBurgh, A. Muñoz, M. R. Zanotelli, C. A. Reinhart-King, *PLoS One* **2019**, *14*, e0216537.
[18] K. P. Mercado, M. Helguera, D. C. Hocking, D. Dalecki, *Tissue Eng. Part C Methods* **2015**, *21*, 671.
[19] C. A. R. Jones, L. Liang, D. Lin, Y. Jiao, B. Sun, *Soft Matter* **2014**, *10*, 8855.
[20] M. G. McCoy, B. R. Seo, S. Choi, C. Fischbach, *Acta Biomater.* **2016**, *44*, 200.
[21] E. E. Antoine, P. P. Vlachos, M. N. Rylander, *PLoS One* **2015**, *10*, e0122500.
[22] M. Achilli, D. Mantovani, *Polymers (Basel).* **2010**, *2*, 664.
[23] Y. Yang, L. M. Leone, L. J. Kaufman, *Biophys. J.* **2009**, *97*, 2051.
[24] A. P. G. Castro, P. Laity, M. Shariatzadeh, C. Wittkowske, C. Holland, D. Lacroix, *J. Mater. Sci. Mater. Med.* **2016**, *27*, 79.
[25] V. B. Shenoy, H. Wang, X. Wang, *Interface Focus* **2016**, *6*, 20150067.
[26] E. Bar-Kochba, J. Toyjanova, E. Andrews, K.-S. Kim, C. Franck, *Exp. Mech.* **2015**, *55*, 261.
